# Characterization of the Small RNA Transcriptomes of Androgen Dependent and Independent Prostate Cancer Cell Line by Deep Sequencing

**DOI:** 10.1371/journal.pone.0015519

**Published:** 2010-11-30

**Authors:** Gang Xu, Jinyu Wu, LingLin Zhou, Binghua Chen, Zhongsheng Sun, Fangqing Zhao, Zhihua Tao

**Affiliations:** 1 Center for Clinical Laboratory Diagnosis, the First Affiliated Hospital of Wenzhou Medical College, Wenzhou, China; 2 Institute of Genomic Medicine, Wenzhou Medical College, Wenzhou, China; 3 Behavioral Genetics Center, Institute of Psychology, Chinese Academy of Science, Beijing, China; 4 Beijing Institutes of Life Science, Chinese Academy of Science, Beijing, China; Queensland University of Technology, Australia

## Abstract

Given the important roles of miRNA in post-transcriptional regulation and its implications for cancer, characterization of miRNA facilitates us to uncover molecular mechanisms underlying the progression of androgen-independent prostate cancer (PCa). The emergence of next-generation sequencing technologies has dramatically changed the speed of all aspects of sequencing in a rapid and cost-effective fashion, which can permit an unbiased, quantitive and in-depth investigation of small RNA transcriptome. In this study, we used high-throughput Illumina sequencing to comprehensively represent the full complement of individual small RNA and to characterize miRNA expression profiles in both the androgen dependent and independent Pca cell line. At least 83 miRNAs are significantly differentially expressed, of which 41 are up-regulated and 42 are down-regulated, indicating these miRNAs may be involved in the transition of LNCaP to an androgen-independent phenotype. In addition, we have identified 43 novel miRNAs from the androgen dependent and independent PCa library and 3 of them are specific to the androgen-independent PCa. Function annotation of target genes indicated that most of these differentially expressed miRNAs tend to target genes involved in signal transduction and cell communication, epically the MAPK signaling pathway. The small RNA transcriptomes obtained in this study provide considerable insights into a better understanding of the expression and function of small RNAs in the development of androgen-independent prostate cancer.

## Introduction

Prostate cancer (PCa) is the most common malignancy of the male genitourinary tract and the third leading cause of cancer death [1], [2]. As PCa growth is initially dependent on androgens for survival, androgen deprivation therapy (ADT) has been the mainstay of treatment for PCa. However, these tumors will eventually progress to an androgen-independent phenotype and fail to respond to ADT treatment, becoming the major obstacle of clinical therapy. Understanding the molecular mechanisms that underlie the progression of androgen-independent PCa will shed considerable lights on possible treatment strategies for PCa. miRNAs (microRNAs) are small, non-coding RNA (∼20–22 nucleotides) that negatively regulate gene expression at the post-transcriptional level [3]. Accumulating evidences indicate that miRNAs may function as tumor suppressors and oncogenes [4], [5]. Given the important roles of miRNAs in post-transcriptional regulation, identification of these differentially expressed and novel miRNAs will facilitate us to uncover the molecular mechanisms underlying the progression of androgen-independent prostate cancer.

To characterize the small RNA transcriptome, the new ‘deep-sequencing’ technologies, such as Roche 454 and Illumina Solexa, have been employed which have significant advantages over previous hybridization-based methodologies, such as microarray and PCR-based assays [6], [7]. Firstly, it provides a more integrated view of the miRNAs transcriptome. High-throughput sequencing has the ability to identify modest or even low abundant miRNAs exhibiting expression differences between distinct samples, to an extent that previously could not be effectively detected. Secondly, direct sequencing also offers the potential to detect the length variation of mature miRNA and possible enzymatic modifications. Thirdly, high-throughput sequencing allows the successful discovery of novel miRNAs, which need not rely on querying candidate regions of the genome but rather can be achieved by direct observation and validation of the folding potential of flanking genomic sequence. Taken together, next-generation sequencing technologies offer a highly robust, accurate and scalable system that sets a new standard for rapid, productive and cost-effective investigation of miRNA transcriptome.

Till now, there are six genome-wide miRNA expression studies in prostate cancer as reviewed by Gandellini et al. [8]. The first miRNA expression profiling of prostate cancer was performed by Lu and colleagues [5], in which they used a bead-based flow cytometric technique to evaluate the miRNA profiling in different tumor types. The remaining five studies applied microarray or bead-based hybridization method to investigate miRNA expression signatures in prostate cancer compared with normal tissue, and identified a number of aberrant expressed miRNAs in tumor cells. However, these studies only focused on the comparison of miRNA expression profiles between PCa samples and surrounding non-tumor tissues, while not revealing what kinds of miRNA might be correlated with the transition from androgen-sensitive to androgen-independent in prostate cancer. Most recently, Sun et al. found that several miRNAs, in particular miR-221 and miR-222, were significantly over-expressed in androgen-independent prostate cancer cells compared with those in the androgen-dependent cell line, and implied the involvement of both miRNAs in the development and progression of the androgen-independence of prostate cancer [9]. deVere White et al. (2009) identified a small set of miRNAs were aberrantly expressed in androgen-independent PCa cell lines using microarray technology [10]. A detailed comparison of miRNA expression profiles between the androgen-dependent and androgen-independent cancers may uncover how the miRNA pathway is involved in the progression of prostate cancer to androgen independence. Using the next-generation sequencing, we have obtained miRNA expression signatures in androgen-independent prostate cancer and identified 83 miRNAs differentially expressed in LNCaP-AI cell lines, as well as putative targets for these miRNAs. To our knowledge, this study represents the first example of small RNA transcriptome by high-throughput sequencing in the prostate cancer and has demonstrated that deep sequencing can serve as an ideal, rapid and cost-effective platform for characterization of small RNA expression profiles.

### Materials and Methods

### Cell Culture

The androgen-dependent LNCaP cell line was obtained from American Type Culture Collection (Rockville, MD) and was routinely maintained in a regular medium: phenol red-positive F-12 medium (Gibco) with 10% FBS (Biowest) at 37°C in 5% CO_2_. Cells were fed twice per week and split once per week with trypsinization (defined as one passage). To establish an androgen-independent cell line, LNCaP was first maintained in phenol red-free DMEM/F-12 medium (Gibco) with 10% charcoal/dextran-treated FBS (Biowest). After cultured for 5 weeks, cells were transferred to the above medium with 1.0×10^−7^ mol/L flutamide (Schering Plough) and cultured for another 105 passages.

### Cell Proliferation Assay

Cells were seeded at a density of 3×10^3^ cells/well in a 96-well culture plate in 90 µl regular media. After 24 hours of incubation at 37°C in 5% CO_2_, the culture medium was changed with half of the wells receiving regular medium and half receiving androgen free medium (phenol red-free medium with 5.0×10^−6^ mol/L flutamide). The cells were incubated in 5% CO_2_ at 37°C for 72 hours, 10 µl of CCK-8 solution (Dojindo) was added to each well, followed by incubation for 3 hours at 37°C, and the absorbance was finally determined at 450 nm using microplate reader (Bio-Rad).

Western blotting was carried out by the methods described previously [11]. The following antibodies were used: rabbit anti-Bcl-2 (1∶200, Cell Signaling), rabbit anti-Bax (1∶100, Cell Signaling) and mouse anti-β-actin (1∶1000, Cell Signaling).

### Cell Cycle Assay

Cells were suspended in hormone-free and regular medium,respectively, and were then planted in three 24-well plates with a number of 3.3×10^5^ cells per well. After incubation in 5% CO_2_ at 37°C for 24 hours, flutamide was added into the holes. The cells were harvested after incubating in 5% CO_2_ at 37°C for 72 hours. The pretreatment procedure of cell cycle analysis was following the manual of Cycle Test^plus^DNA Reagent Kit (Becton Dickinson). Cell cycle analysis was performed using a FACScan flow cytometer (BD Company).

### Real-time RT-PCR

Small RNAs (<200 nt) were isolated with *mirVana*™ PARIS ™ Kit (Ambion) according to the manufacturer's instructions. For RT reactions, 1 µg of small RNA was used for reverse transcription with miScript Reverse Transcription Kit (Qiagen), performed at 37°C for 60 min and a final incubation at 95°C for 5 min. MiRNA real-time RT-PCR was carried out by using the miScript SYBR Green PCR kit (Qiagen) on an Applied Biosystems 7000 real-time PCR machine (ABI). The PCR reaction was conducted at 95°C for 15 min, followed by 40 cycles of incubation at 94°C for 15 s, 55°C for 30 s, and 70°C for 30 s. Each PCR was repeated at least three times. The relative expression level of each miRNA was normalized by against U6 snRNA levels. Fold-change was calculated according to the 2^−ΔΔCt^ method.

### Small RNA Library Construction and High-throughput Sequencing

The isolated total RNA was size-fractionated on a 15% tris-borate-EDTA (TBE) urea polyacrylamide gel to enrich for molecules of 15–30 nt. The small RNA was ligated with 3′ (5′-pUCGUAUGCCGUCUUCUGCUUGidT-3′) and 5′ (5′-GUUCAGAGUUCUACAGUCCGACGAUC-3′) adapters using T4 RNA ligase and was again size-fractionated on a 15% TBE urea polyacrylamide gel. The resulting RNA was reversely transcribed to cDNA with Solexa's small RNA RT-Primer (5′-CAAGCAGAAGACGGCATACGA-3′). The cDNA was used as a template for PCR amplification using Solexa's small RNA primer set (5′-CAAGCAGAAGACGGCATACGA-3′;5′-AATGATACGGCGACCACCGACAGGTTCAGAGTTCTACAGTCCGA-3′). Finally, approximately 20 µg of small RNA were used for sequencing using Illumina 1 G Genome Analyzer according to the manufacturer's protocols.

### Read Filter and Small RNA Annotation

For deep-sequencing reads produced by Illumina Genome Analyzer, low quality reads were filtered out to exclude those most likely to represent sequencing errors and 3/5′ adaptor sequences were subsequently trimmed into clean full length reads formatted into a non-redundant Fasta format. The occurrences of each unique sequence reads were counted as sequence tags (the number of reads for each tag reflects relative expression level) and only small RNA sequences of 18 to 30 nt were retained for further analysis.

All unique sequence tags that pass above filters were mapped onto the reference human genome using the SOAP 2.0 program with at most two mismatches [12]. Subsequently, the unique sequence tags were aligned against miRBase14.0, computationally predicted human ncRNAs and Rfam 9.1 (http://rfam.sanger.ac.uk/), the RepeatMasker annotation from RepBase 14.09 (http://www.girinst.org/), the human genes UCSC annotation hg18 (http://genome.ucsc.edu/) to classify known miRNA, degradation fragments of non-coding RNA, genomic repeats and mRNA, respectively. Sequences that did not overlap with any of these annotations but could be aligned to the reference genome were termed “unclassified”.

### Differential Expression Detection and Novel miRNA Prediction

To compare differentially expressed miRNAs between LNCaP and LNCaP-AI, read counts of each identified miRNAs was normalized to the total number of miRNA reads. The statistical significance (P-value) was inferred based on the Bayesian method, which was developed for analysis of digital gene expression profiles and could account for the sampling variability of tags with low counts [13]. A specific miRNA was deemed to be significantly differentially expressed if the *P* value given by this method was ≤0.001 and there was at least a 2-fold change in normalized sequence counts. The target genes for each differentially expressed miRNA were predicted using TargetScan (http://www.targetscan.org/), miRanda (http://www.microrna.org/), PicTar (http://pictar.mdc-berlin.de/) and RNAhybrid (http://bibiserv.techfak.uni-bielefeld.de/rnahybrid/). Given that miRNA targets prediction often suffer from high level of false positives, only the target gene supported by at least three independent tools were taken into account. The Gene Ontology (GO) terms and KEGG pathways of targeted genes were annotated using the DAVID gene annotation tool (http://david.abcc.ncifcrf.gov/UTH).

The unannotated sequences that could be mapped to the human genome were considered for detecting candidate novel miRNA genes. In brief, 100 nucleotides of genomic sequence flanking each side of these sequences were extracted and the RNA secondary structures were predicted using RNAfold (http://rna.tbi.univie.ac.at/cgi-bin/RNAfold.cgi). Candidate novel miRNA was identified using MIREAP under default settings, which has been widely adopted in related studies [14], [15].

All sequencing data along with annotation results can be available at http://59.79.168.90/pca.

## Results

### Establishment of an Androgen-independent LNCaP Cell Line

**Figure 1:**
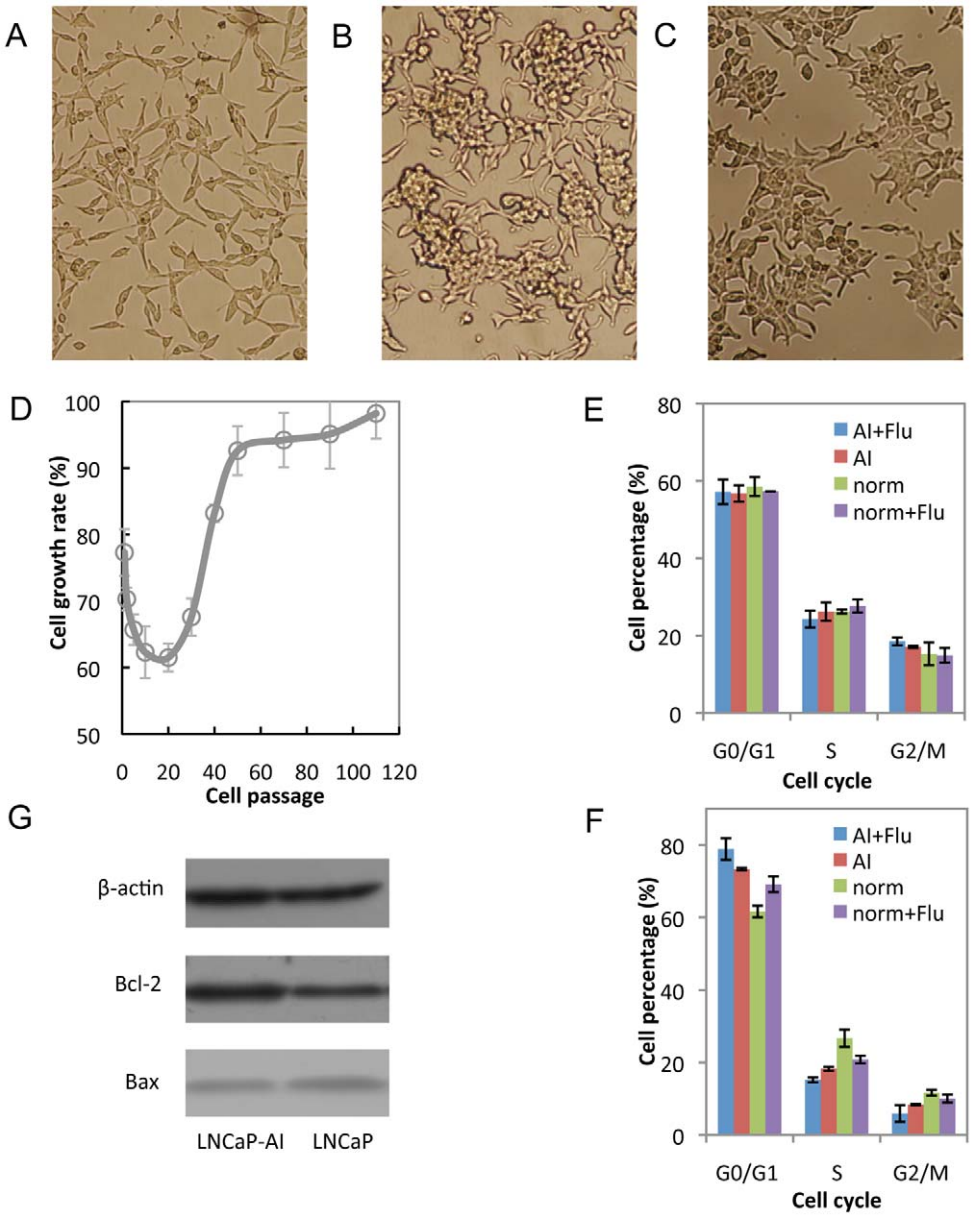
Changes in the biological characteristics of LNCaP cell line from androgen-dependent to androgen-independent. (A) The morphology of parental LNCaP cells, which were cultured in regular medium for 3 days. (B) Neuroendocrine phenotype of LNCaP cells. LNCaP cells were maintained in androgen-depleted medium and cultured for 3 passages. (C) Morphology of LNCaP-AI cells. LNCaP cells were cultured with flutamide in androgen-depleted medium for 110 passages. (D) The cell growth rates of androgen-depleted cultured LNCaP cells at different passages when grown in androgen deprivation environment. (E–F) The effects of androgen-depleted environment and flutamide on LNCaP-AI (E) and LNCaP-FGC (F) cell cycle. (AI: androgen-depleted medium; Flu: 1.0×10^−7^ mol/L of flutamide; norm: regular medium). (G) Bcl-2 and Bax expression in LNCaP and LNCaP-AI cells.

Based upon the reports that androgen sensitive LNCaP cells (Fig. 1A) can be converted to androgen-independent cells [16], [17], we attempted to generate an androgen-independent cell line by continuously culturing of LNCaP cells *in vitro*. After three weeks culture initiation in hormone-deprived medium, the proliferation of the cells gradually slow down and a considerable number of cells (about 40–50%) underwent apoptosis (Fig. 1B). The remaining ones changed in morphology to display a neuroendocrine-like phenotype (Fig. 1B). With increasing passage number, the cells appeared with obvious morphological variations and become small and flat, developing the ability to grow in an androgen-independent fashion (a phenotype that we designated as LNCaP-AI) (Fig. 1C). To estimate the effects of androgen-free environment on LNCaP-AI cells growth, we continuously examined the cell proliferation rate of the cell line at different passages. It is shown that cell growth rate of LNCaP-AI cells was shown a gradual downtrend from passage 1, 2, 5, 10 to 20, however, as the passage number increased, cell proliferation rate was increased gradually and almost reached to 100% (Fig. 1D). After 110 passages during 2 years of culture, cell growth was stabilized and can grow well in androgen-depleted environment.

To further prove that the LNCaP-AI cells have acquired the ability to adapt to the hormone-free environment, more studies are needed for insights into the effects of flutamide or androgen-free environment on the cell cycle of LNCaP cells by flow cytometry, and the androgen-dependent LNCaP cells were included as a control. As expected, flutamide or androgen-free environment had no significant effect on cell cycle distribution in the LNCaP-AI cells (Fig. 1E). In contrast, this treatment in the LNCaP cells was induced an accumulation of cells in the G0/G1 phase and accompanied with a decrease in the S phase, and the inhibition effect was much stronger when both of them were combined (Fig. 1F). Previous study has revealed that Bcl-2 is overexpressed in the progression to androgen-refractory prostate cancer [1]. To characterize the expression of both Bcl-2 and Bax (Bcl-2–associated X protein) proteins in the two cell lines (LNCaP and LNCaP-AI), Western blotting analysis was performed (Fig. 1G). We found an increased expression of Bcl-2 protein in LNCaP-AI cells in comparison with LNCaP cells. Meanwhile, LNCaP cells expressed a similar level of Bax protein. These results suggest that LNCaP-AI cells gained an enhanced anti-apoptotic activity. Based on the above observations, we have successfully established an androgen-independent LNCaP cell line through long-term culture of LNCaP cells.

### Small RNA Transcriptomes and Annotation

**Figure 2:**
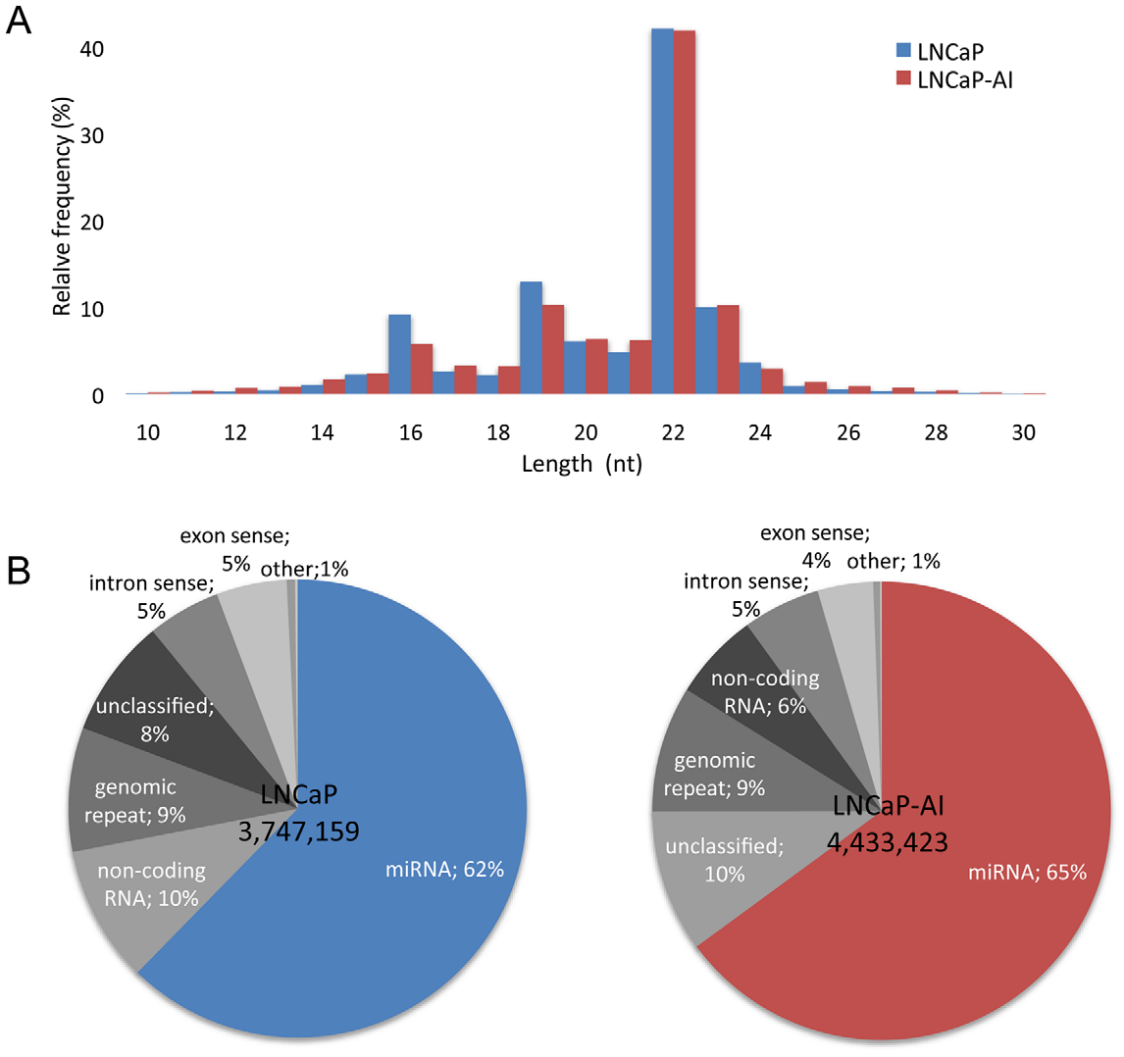
Size distribution and annotation of small RNAs from the libraries of LNCaP and LNCaP-AI. (A) Length distribution of sequenced reads. Both libraries accumulated 22-nucleotide small RNAs, which is consistent with the typical size of miRNAs. B) The proportions of various classes of small RNAs detected in LNCaP and LNCaP-AI. It is indicated that the majority of reads in both libraries belonging to the miRNA families.

To investigate small RNA transcriptomes, Illumina high-throughput sequencing technology was employed to both LNCaP and LNCaP-AI small RNA libraries. Initially, a total of 9,107,833 and 10,083,251 raw sequence reads were produced for LNCaP and LNCaP-AI, respectively. After filter and trimming of the reads with low quality and adaptor, 3,978,524 and 4,734,866 sequence reads were obtained corresponding to 302,325 (LNCaP) and 416,924 (LNCaP-AI) unique tags. Observation the length distribution of small RNAs, we found that the majority of them from the both libraries were 22 nt in size (Fig. 2A), which is consistent with the typical size of miRNA from Dicer digestion products. Meanwhile, there were a high percentage of identical sequence tags (88.90%) between LNCaP and LNCaP-AI. These results indicated that miRNAs have been successively enriched from both libraries.

Subsequently, the 18–30 nt sequences from both libraries were selected to map to the human genome, and a total of 3,747,159 (94.18%) and 4,433,423 (93.63%) sequences were detected to match the genome with at most two mismatches, respectively (Fig. 2B). Based on human genome annotations and a number of well-characterized RNA databases (see Materials and Methods), these small RNA sequences were annotated as known miRNA, degradation fragments of non-coding RNA (tRNA, rRNA, snRNA/snoRNA et al.), genomic repeat, mRNA or unclassified. As expected, the most abundant RNA category from both libraries was known miRNAs: 65.22% for LNCaP and 62.33% for LNCaP-AI. The remaining less abundant categories were non-coding RNA and genomic repeats. In addition, a small proportion of reads could be mapped to coding sequences, which are likely to be RNA degradation products. Accumulating evidence indicated that some genomes can also transcribe short functional non-coding transcripts, such as endogenous small interfering RNAs or repeat-associated interfering RNAs, which has been characterized to regulate of retrotransposition repression [18]. For example, it is revealed that L1 retrotransposition is suppressed by endogenously encoded small interfering RNAs in human cultured cells. Thus, it is noteworthy that these small RNAs may also contain important biological function that deserves further attention.

### Differentially Expressed miRNA Between LNCaP and LNCaP-AI

Based on miRBase, 360 miRNAs (285 miRNAs and 75 miRNAs*) and 380 miRNAs (282 miRNAs and 98 miRNAs*) were detected in LNCaP and LNCaP-AI, respectively (Table S1). First, we found that different miRNAs exhibited significantly different expression levels as measured by the frequency of read counts, indicating a remarkable functional divergence of these miRNAs. In LNCaP-AI, for example, about 8.99% of miRNAs and miRNA*s are at high read counts (>1000), among which the hsa-let-7 family (hsa-let-7c, hsa-let-7f, hsa-let-7a, hsa-let-7d, hsa-let-7b and hsa-let-7e) is one of the most abundant miRNAs in our data set, contributing >8.94% of the total miRNA reads. miRNA count within the ranges of intermediate 100–1000 reads were up to 17.4% and the remaining miRNA counts for approximately 73.7%.

**Figure 3:**
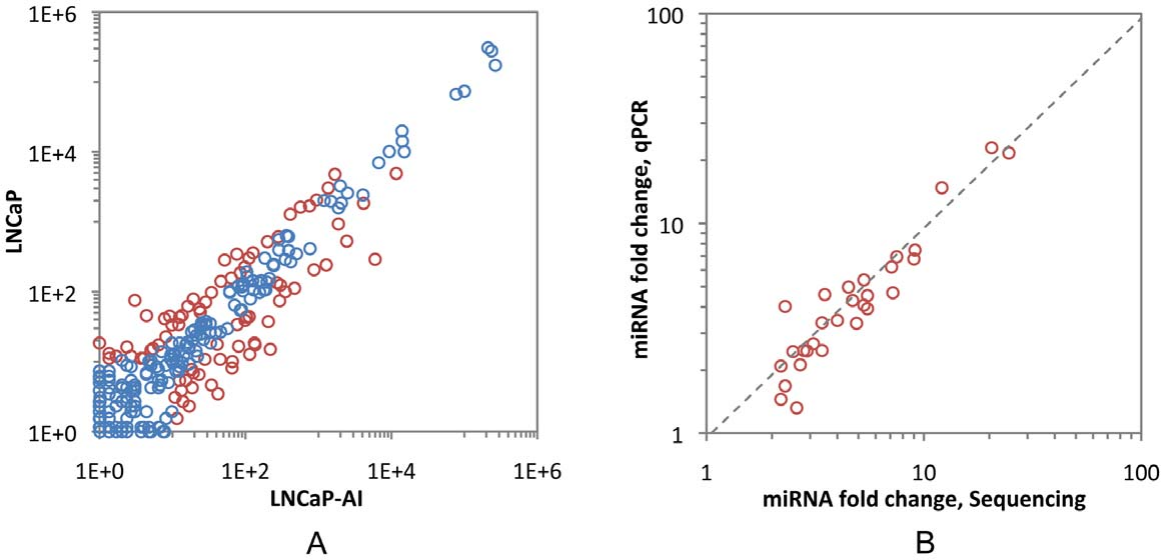
The miRNAs expression level and validation of the robust of Solexa sequencing with qRT-PCR analysis. (A) Expression level of LNCaP and LNCaP-AI miRNAs. The count of each miRNA was plotted after normalization. Red color circle shows differentially expressed miRNAs between LNCaP and LNCaP-AI with at least a 2-fold change and *p*-value below 0.001. (B) Solexa vs. qRT-PCR of miRNA fold-changes.

The relative count of sequencing reads could be used to quantify miRNA expression levels between LNCaP and LNCaP-AI. Based on the normalized number of reads per sample (specifc miRNA/total sequencing tags in the library), the majority of miRNAs (256) were expressed approximately equally between the two libraries. However, 83 of them were identified to be differentially expressed with a fold changes >2.0 and *P*-value <0.001 (Table 1, Fig. 3A and Table S1). Among them, 41 were up-regulated and 42 were down-regulated. To further validate these differentially expressed miRNAs, 29 individual miRNAs were selected to perform quantitative RT-PCR assay from independent biological replicates. These 29 selected miRNAs covered both highly expressed miRNAs (miR-222, miR-30a*, miR-100, miR-10b, miR-148b, miR-1323, miR-221, miR-7, miR-223, miR-374b, miR-486-5p, miR-7a*, miR-125b-2, miR-27a and miR-423-5) and lower expressed miRNAs (miR-15b, miR-21, miR-17, miR-28-5p, miR-532-3p, miR-200c, miR-93, miR-96, miR-200b*, miR-331-3p, miR-200b, miR-106a, miR-301b and miR-301a). As shown in Fig. 3B, a strong correlation (Pearson's correlation  = 0.91) was revealed between the Illumina deep sequencing data and the quantitative RT-PCR measurements, indicating the robustness of deep sequencing based expression analysis.

**Table 1:** Top 20 miRNAs differentially expressed between the LNCaP and LNCaP-AI libraries.

miRNA ID	Relative count		P-value	Fold change
	LNCaP	LNCaP-AI		
hsa-miR-222	290.95	5977.41	0	20.54
hsa-miR-124	15.02	219.24	5.93E-127	14.59
hsa-miR-30a*	3.46	42.02	3.84E-24	12.11
hsa-miR-100	12.71	114.53	6.86E-57	9.01
hsa-miR-30c-2*	8.09	65.40	1.29E-31	8.08
hsa-miR-30a	17.34	135.20	1.04E-62	7.80
hsa-miR-10b	1.54	11.52	2.31E-06	7.47
hsa-miR-296-3p	4.62	34.22	1.58E-16	7.40
hsa-miR-629	2.31	16.94	9.28E-09	7.33
hsa-miR-148b	18.49	132.83	3.46E-59	7.18
hsa-miR-760	10.02	66.08	6.76E-29	6.59
hsa-miR-1323	37.38	205.69	1.22E-77	5.50
hsa-miR-200b	284.02	51.84	1.09E-109	5.48
hsa-miR-96*	16.18	2.37	1.83E-08	6.82
hsa-miR-106a	11.95	1.69	1.19E-06	7.05
hsa-miR-362-5p	11.18	1.36	1.06E-06	8.25
hsa-miR-301b	13.11	1.36	4.15E-08	9.67
hsa-miR-1277	45.47	4.41	1.09E-25	10.32
hsa-miR-664	18.50	1.00	3.72E-15	18.50
hsa-miR-301a	75.15	3.05	2.34E-52	24.64

### Novel miRNA Genes

One of the most important advantages for high-throughput sequencing of small RNA transcriptome is that it allows to discovery of potential novel miRNAs. To identify candidate novel miRNAs in LNCaP and LNCaP-AI, we first filtered out Illumina sequence tags that have been classified into annotated categories, such as known miRNAs, non-coding RNA, genomic repeats or coding sequences. We found that 235,058 and 259,454 of the small RNA sequence tags in LNCaP and LNCaP-AI, respectively, were derived from unannotated regions of the human genome. These unclassified tags along with 100-bp flanking sequences were analyzed by MIREAP, a sophisticated tool commonly used to identify novel miRNAs from high-throughput sequencing data [14], [15]. In this way, we identified 43 putative novel miRNAs from both libraries (Table 2 and Table S2): 22 in LNCaP and 31 in LNCaP-AI (Table 2 and Table S2), and only 10 of them are shared by both libraries.

**Table 2:** The novel miRNAs validated by qRT-PCR.

miRNA ID	Sequence	Length (nt)	Genomic location	5′/3′ arm	Precursor length (nt)	MFE	Relative count	
							LNCaP	LNCaP-AI
hsa-novel-miR-01*	TCGGGCGGGAGTGGTGGCTTTT	22	chr6:28918819:28918903:+	3′	85	−22.2	277.47	816.64
hsa-novel-miR-02*	ACTGACAGGAGAGCATTTTGA	21	chr5:89312448:89312527: −	3′	80	−39.3	0	45.41
hsa-novel-miR-03*	TCTCAGGAGTAAAGACAGAGTT	22	chr11:70718384:70718464: −	3′	81	−49.2	0	21.69
hsa-novel-miR-04*	GCAAATGATGTGAGAGATTC	20	chr6:168343859:168343952:+	3′	94	−20.2	0	11.52
hsa-novel-miR-05*	AAAAGCTGGGTTGAGAGGGTAA	22	chr18:21901636:21901711:+	3′	76	−32.6	4.62	11.18
hsa-novel-miR-06*	ACTGGACTTGGAGTCAGAAGA	21	chr10:132760891:132760988: −	3′	98	−34.9	10.02	10.17
hsa-novel-miR-07*	TCTGATGATGATGATGGTGCT	21	chr15:89826590:89826677:+	5′	88	−18.2	24.66	0
hsa-novel-miR-08*	CAAAATGATGAGGTACCTGATA	22	chr20:3194751:3194835:+	5′	85	−20.4	73.22	0
hsa-novel-miR-09	ACCCCAGGATGCCAGCATAGTT	22	chr5:138611933:138612022:+	5′	90	−32.2	19.65	0
hsa-novel-miR-10	TAGCTCTGATGATGGTGGTTTCT	23	chr5:176878879:176878970: −	3′	92	−23.2	11.18	0

*indicates that the miRNA has been successfully validated by qRT-PCR. MFE refers to the minimum free energy.

We also observed that these 43 miRNAs have a size range of 20–24 nt and the lengths of their predicted hairpin structures vary from 65–98 nt, which is similar to known human miRNAs (Table S2). The minimum free energies of their precursors range from −18.20 to −67.40 kcal/mol with the average value of −52.70 kcal/mol. To further validate these novel miRNAs, we performed real-time RT-PCR analysis on all of 10 novel miRNA candidates with a normalized sequencing frequency larger than 10 (the others were excluded in view of their low expression level and the sensitivity of real-time RT-PCR analysis). As a result, we have successfully validated eight of them (Table 2), indicating that 80% could be validated by a sequencing-independent method.

In comparison with other miRNAs in our study, these novel miRNAs have a much lower expression level with an average normalized sequencing frequency of 45, indicating that the majority of them may not exhibit significant functions in prostate cancer. Among them, three LNCaP-AI specific miRNAs exhibited relative sequence counts large than 10 and validated by real-time RT-PCR, implicating their likely involvement in the development of LNCaP-AI, and thus deserve further functional evaluation.

### Predicted Targets of Differentially Expressed miRNAs

To explore the biologic function of the differentially expressed miRNAs identified in our analysis, we performed computational analysis using four independent algorithms, including TargetScan, miRanda, RNAhybrid and PicTar, for identification of predicted messenger RNA targets for each miRNA to be significantly differentially expressed. Table S3 lists predicted targets that were supported by at least three of the above four algorithms, in which a total of 6902 genes were potential targets of these miRNAs. Interestingly, among these predicted targets, AKT3 was involved in 11 significantly down-expressed miRNAs. AKT3 encodes a member of serine/threonine protein kinase family, which is known to be involved in a wide variety of biological processes including cell proliferation, differentiation, apoptosis, and tumorigenesis. The up-expression of AKT3 in androgen-independent prostate cancer cell lines reveals that it may contribute to the more aggressive phenotype of androgen-independent prostate carcinomas [19].

To evaluate target gene functions, we annotated these predicated miRNA targets with gene ontology (GO) and KEGG schemes using DAVID. Predicted miRNA targets populated many major GO categories, and for some of them, the number of gene targets was significantly enriched (*P*<0.001, with Benjamini correction). The three GO classifications, molecular function, biological process and cellular component were evaluated by level, but only significant terms at level 5 of biological process are listed in Table S3. As expected, most of the significant GO terms were related to regulation of transcription (e.g. GO:0045449, GO:0006351 and GO:0006357). There are also a number of significantly enriched GO categories, including cell morphogenesis (GO:0000902), intracellular transport (GO:0046907), and apoptosis (GO:0006915). To analyze the role that miRNAs play in the regulatory networks, we assigned putative miRNA targets into KEGG pathways, and identified 14 of them were significantly enriched (*P*<0.001, with Benjamini correction). Most of these miRNAs tend to target genes involved in signal transduction and cell communication (Table S4). For example, we found that 130 target genes (2.3%) could be assigned to the MAPK signaling pathway, which is involved in a wide range of cellular responses, including gene expression, differentiation, proliferation and apoptosis [20]. In prostate cancer, MAPK signaling pathways are generally considered to promote tumor growth and the emergence of hormone-refractory disease [21], [22], [23].

## Discussion

In this study, we have identified a large set of miRNAs that are differentially expressed underlying the progression of androgen-independent prostate cancer, which were also verified by qRT-PCR. Some of these miRNAs are well supported by recently published studies. A striking example comes from the over-expression of miR-221 and miR-222 in the LNCaP-AI sample by a 10-20 fold increase, where both of them are among the most abundant miRNAs in the LNCaP-AI cell line. Overexpression of miR-221 or miR-222 in LNCaP could significantly reduce the level of the dihydrotestosterone induced up-regulation of prostate-specific antigen expression and increase androgen-independent growth of LNCaP cells [9]. Several lines of evidence suggested that miR-221 and miR-222 could down-regulate the tumor suppressor p27 in LNCaP cells, and their overexpression might be one of the factors contributing to the progression of prostate carcinoma [24]. Moreover, increased expression of miR-125b, miR-30c, miR-100 that we observed in LNCaP-AI cell lines was also identified to be up-regulated in five AI CaP cell lines [25], and *in vitro* experiment showed that miR-125b could stimulate the AI growth and down-regulate the expression of BaK1 [25]. The reduction of miR-141, miR-19b, miR-22, miR-29a, and miR-29b identified here was also noted among the 15 miRNAs that were decreased in four hormone-refractory prostate carcinomas as described in Porkka et al.'s microarray-based study [26].

We also identified a set of differentially expressed miRNAs, which has not been documented in prostate tumorigenesis or AI development. For example, miR-124, miR-148b, miR-320a and miR-423-5p were up-regulated by 2 to 14-fold, while miR-29a, miR-93, miR-200 and miR-1277 were down-regulated by 2 to 10-fold, suggesting that these miRNAs could exert a role as tumour suppressor genes or oncogenes in prostate cancer development. It is of interest to note that all five members of the miRNA-200 family (miR-200a, miR-200b, miR-200c, miR-141 and miR-429) were significantly down-regulated in LNCaP-AI cells. All these miRNAs were also identified to be down-expressed in cells that had undergone epithelial to mesenchymal transition [27], which is viewed as an essential early step for progression of non-invasive tumor cells into metastatic carcinomas [28]. Targets of these microRNAs were found to be E-cadherin transcriptional repressors ZEB1 and SIP1, factors previously implicated in tumour metastasis [27]. A most recent study revealed that ZEB1 expression could enhance transendothelial migration in prostate cancer cells [29]. Taken together, these findings indicate down-regulation of the miR-200 family may also contribute to the progression of prostate cancer.

Genome-wide miRNA expression profiling will undoubtedly enhance our understanding of miRNAs and their roles in the process of tumor invasion and metastasis [1], [2]. However, the effort of depicting a clear microRNA profile in prostate cancer is not that conclusive and different studies may give opposite expression of some microRNAs in prostate cancer [4], [8]. Such discrepancies may be due to the application of different profiling methods (e.g. microarray, sequencing, qRT-PCR), specimen collection, miRNA preparation, or analytical tools. Notably, certain miRNAs may act as either oncogenes or tumor suppressors in different contexts, and thus may lead to conflicting expression profiles [30]. In this study, both the direct sequencing and qRT-PCR assays indicated that miR-29a was significantly down-regulated by 2.8-fold in the LNCaP-AI cell line as compared to in the LNCaP cell line. Similar patterns were also found in haematopoietic, cholangiocytic and lung tumours [31], [32], [33], whereas in breast carcinomas miR-21a was over-regulated [30]. Hence it is important to interpret miRNA transcriptome results with caution.

In sum, we have investigated the miRNA expression profiles in human prostate cancer using direct miRNA sequencing approach and qRT-PCR. Unlike previous studies, we focused on how miRNAs response to the development of androgen independence in prostate cancer. We found a number of miRNAs were significantly differentially expressed between two different phenotypes of prostate cancer, and some of them are well consistent with previous findings. Although miRNA expression in induced cell lines is not necessarily the same as clinical samples, we believe that the differentially expressed miRNA identified in this study would provide good candidates for further functional evaluation.

## Supporting Information

Table S1 **The known miRNAs expressed in LNCaP and LNCaP-AI libraries.**
(DOC)

Table S2 **The novel miRNAs identified in LNCaP and LNCaP-AI libraries.**
(DOC)

Table S3 **The GO term predicted targets of differentially expressed miRNAs.**
(DOC)

Table S4 **The KEGG term of predicted targets of differentially expressed miRNAs.**
(DOC)
