# Supplementary material for: Characterization of the Small RNA Transcriptomes of Androgen Dependent and Independent Prostate Cancer Cell Line by Deep Sequencing
Source: PLoS One. 2010 Nov 30;5(11):e15519. doi: 10.1371/journal.pone.0015519 (PMC2994876; doi:10.1371/journal.pone.0015519)
Supplement: Table S1 — The known miRNAs expressed in LNCaP and LNCaP-AI libraries. (DOC) [file pone.0015519.s001.doc]

**Table S1. The known miRNAs expressed in LNCaP and LNCaP-AI libraries**

| miRNA ID | Original sequence | LNCaP | | | LNCaP-AI | | | P-value | Fold change | Significant |
| --- | --- | --- | --- | --- | --- | --- | --- | --- | --- | --- |
|  |  | 5'/3'  arm | Most abundant sequence | Relative  count | 5'/3'  arm | Most abundant sequence | Relative  count |  |  |  |
| hsa-miR-222 | AGCTACATCTGGCTACTGGGT | 3' | AGCTACATCTGGCTACTGGGTC | 290.95 | 3' | AGCTACATCTGGCTACTGGGTC | 5977.41 | 0 | 20.54 | ** |
| hsa-miR-124 | TAAGGCACGCGGTGAATGCC | 3' | TAAGGCACGCGGTGAATG | 15.02 | 3' | TAAGGCACGCGGTGAATG | 219.24 | 5.93E-127 | 14.59 | ** |
| hsa-miR-30a* | CTTTCAGTCGGATGTTTGCAGC | 3' | CTTTCAGTCGGATGTTTGCAG | 3.46 | 3' | CTTTCAGTCGGATGTTTGCAG | 42.02 | 3.84E-24 | 12.11 | ** |
| hsa-miR-100 | AACCCGTAGATCCGAACTTGTG | 5' | AACCCGTAGATCCGAACTTGTG | 12.71 | 5' | AACCCGTAGATCCGAACTTGTG | 114.53 | 6.86E-57 | 9.01 | ** |
| hsa-miR-30c-2* | CTGGGAGAAGGCTGTTTACTCT | 3' | CTGGGAGAAGGCTGTTTACT | 8.09 | 3' | CTGGGAGAAGGCTGTTTACT | 65.40 | 1.29E-31 | 8.08 | ** |
| hsa-miR-30a | TGTAAACATCCTCGACTGGAAG | 5' | TGTAAACATCCTCGACTGGAAGC | 17.34 | 5' | TGTAAACATCCTCGACTGGAAGC | 135.20 | 1.04E-62 | 7.80 | ** |
| hsa-miR-1246 | AATGGATTTTTGGAGCAGG | 5' | AATGGATTTTTGGAGCAG | 1 | 5' | AATGGATTTTTGGAGCAGG | 7.79 | 6.49E-06 | 7.79 |  |
| hsa-miR-16-2* | AATGGATTTTTGGAGCAGG | 5' | AATGGATTTTTGGAGCAG | 1 | 5' | AATGGATTTTTGGAGCAGG | 7.79 | 5.31E-07 | 7.79 |  |
| hsa-miR-10b | TACCCTGTAGAACCGAATTTGTG | 5' | TACCCTGTAGAACCGAATTTGT | 1.54 | 5' | TACCCTGTAGAACCGAATTTGT | 11.52 | 2.31E-06 | 7.47 | ** |
| hsa-miR-181a | AACATTCAACGCTGTCGGTGAGT | 5' | AACATTCAACGCTGTCGGTGA | 1 | 5' | AACATTCAACGCTGTCGGTGAG | 7.45 | 7.20E-05 | 7.45 |  |
| hsa-miR-338-3p | AACATTCAACGCTGTCGGTGAGT | 5' | AACATTCAACGCTGTCGGTGA | 1 | 5' | AACATTCAACGCTGTCGGTGAG | 7.45 | 9.98E-07 | 7.45 |  |
| hsa-miR-296-3p | GAGGGTTGGGTGGAGGCTCTCC | 3' | GAGGGTTGGGTGGAGGCT | 4.6245 | 3' | GAGGGTTGGGTGGAGGCT | 34.22 | 1.58E-16 | 7.40 | ** |
| hsa-miR-629 | TGGGTTTACGTTGGGAGAACT | 5' | TGGGTTTACGTTGGGAGAA | 2.3122 | 5' | TGGGTTTACGTTGGGAGA | 16.94 | 9.28E-09 | 7.33 | ** |
| hsa-miR-148b | TCAGTGCATCACAGAACTTTGT | 3' | TCAGTGCATCACAGAACT | 18.4979 | 3' | TCAGTGCATCACAGAACT | 132.83 | 3.46E-59 | 7.18 | ** |
| hsa-miR-760 | CGGCTCTGGGTCTGTGGGGA | 3' | CGGCTCTGGGTCTGTGGGGAG | 10.0197 | 3' | CGGCTCTGGGTCTGTGGGGAG | 66.08 | 6.76E-29 | 6.59 | ** |
| hsa-let-7b* | CTATACAACCTACTGCCTTCCC | 3' | CTATACAACCTACTGCCTTCC | 1.1561 | 3' | CTATACAACCTACTGCCTTC | 6.78 | 0.000855841 | 5.86 |  |
| hsa-miR-501-3p | AATGCACCCGGGCAAGGATTCT | 3' | AATGCACCCGGGCAAGGATT | 1.1561 | 3' | AATGCACCCGGGCAAGGATT | 6.78 | 0.000855841 | 5.86 |  |
| hsa-miR-129-3p | AAGCCCTTACCCCAAAAAGCAT | 3' | AAGCCCTTACCCCAAAAAGCAT | 1.1561 | 3' | AAGCCCTTACCCCAAAAAGCAT | 6.44 | 0.001418082 | 5.57 |  |
| hsa-miR-1323 | TCAAAACTGAGGGGCATTTTCT | 5' | TCAAAACTGAGGGGCATTTTCT | 37.3812 | 5' | TCAAAACTGAGGGGCATTTTCT | 205.69 | 1.22E-77 | 5.50 | ** |
| hsa-miR-221 | AGCTACATTGTCTGCTGGGTTTC | 3' | AGCTACATTGTCTGCTGGGTTT | 241.629 | 3' | AGCTACATTGTCTGCTGGGTTTC | 1281.21 | 0 | 5.30 | ** |
| hsa-miR-1979 | CTCCCACTGCTTCACTTGACTA | 5' | CTCCCACTGCTTCACTTGACTAG | 1.5415 | 5' | ACTCCCACTGCTTCACTT | 8.13 | 0.000385668 | 5.28 |  |
| hsa-miR-143 | TGAGATGAAGCACTGTAGCTC | 3' | GAGATGAAGCACTGTAGCT | 2.6976 | 3' | TGAGATGAAGCACTGTAGCTC | 13.89 | 3.19E-06 | 5.15 | ** |
| hsa-miR-516b | ATCTGGAGGTAAGAAGCACTTT | 5' | CCATCTGGAGGTAAGAAGCACTTT | 1.9269 | 5' | ATCTGGAGGTAAGAAGCACTTT | 9.83 | 0.000106399 | 5.10 |  |
| hsa-miR-618 | AAACTCTACTTGTCCTTCTGAGT | 5' | AAACTCTACTTGTCCTTCT | 1.9269 | 5' | AAACTCTACTTGTCCTTC | 9.83 | 0.000106399 | 5.10 |  |
| hsa-miR-1244 | AAACTCTACTTGTCCTTCTGAGT | 5' | AAACTCTACTTGTCCTTCT | 1 | 5' | AAACTCTACTTGTCCTTC | 5.08 | 8.26E-05 | 5.08 |  |
| hsa-miR-7 | TGGAAGACTAGTGATTTTGTTGT | 5' | TGGAAGACTAGTGATTTTGTTGTT | 16.571 | 5' | TGGAAGACTAGTGATTTTGTTGTT | 80.65 | 6.98E-29 | 4.87 | ** |
| hsa-miR-199a-5p | CCCAGTGTTCAGACTACCTGTTC | 5' | CCCAGTGTTCAGACTACCT | 1 | 5' | CCCAGTGTTCAGACTACCT | 4.74 | 0.005324316 | 4.74 |  |
| hsa-miR-223 | CCCAGTGTTCAGACTACCTGTTC | 5' | CCCAGTGTTCAGACTACCT | 1 | 5' | CCCAGTGTTCAGACTACCT | 4.74 | 0.000155281 | 4.74 |  |
| hsa-let-7i | TGAGGTAGTAGTTTGTGCTGTT | 5' | TGAGGTAGTAGTTTGTGCT | 527.9613 | 5' | TGAGGTAGTAGTTTGTGCT | 2445.86 | 0 | 4.63 | ** |
| hsa-miR-30c | TGTAAACATCCTACACTCTCAGC | 5' | TGTAAACATCCTACACTCTCAGCT | 4.2391 | 5' | TGTAAACATCCTACACTCTCAGCT | 18.64 | 3.48E-07 | 4.40 | ** |
| hsa-miR-221* | ACCTGGCATACAATGTAGATTT | 5' | ACCTGGCATACAATGTAGATTT | 205.7893 | 5' | ACCTGGCATACAATGTAGATTT | 869.84 | 4.56E-263 | 4.23 | ** |
| hsa-miR-877 | ACCTGGCATACAATGTAGATTT | 5' | ACCTGGCATACAATGTAGATTT | 112.1436 | 5' | ACCTGGCATACAATGTAGATTT | 471.01 | 1.56E-142 | 4.20 | ** |
| hsa-miR-1 | TGGAATGTAAAGAAGTATGTAT | 3' | TGGAATGTAAAGAAGTATGTAT | 10.7905 | 3' | TGGAATGTAAAGAAGTATGTAT | 45.07 | 5.90E-15 | 4.18 | ** |
| hsa-miR-30b | TGTAAACATCCTACACTCAGCT | 5' | TGTAAACATCCTACACTC | 1.1561 | 5' | TGTAAACATCCTACACTCAGCT | 4.74 | 0.016139759 | 4.10 |  |
| hsa-miR-374b | ATATAATACAACCTGCTAAGTG | 5' | ATATAATACAACCTGCTAAGTG | 73.9917 | 5' | ATATAATACAACCTGCTAAGTG | 294.13 | 2.38E-85 | 3.98 | ** |
| hsa-miR-138 | ATATAATACAACCTGCTAAGTG | 5' | ATATAATACAACCTGCTAAGTG | 1 | 5' | ATATAATACAACCTGCTAAGTG | 3.73 | 0.001030627 | 3.73 |  |
| hsa-miR-296-5p | ATATAATACAACCTGCTAAGTG | 5' | ATATAATACAACCTGCTAAGTG | 1 | 5' | ATATAATACAACCTGCTAAGTG | 3.73 | 0.001030627 | 3.73 |  |
| hsa-miR-1308 | GCATGGGTGGTTCAGTGG | 5' | GCATGGGTGGTTCAGTGG | 99.8117 | 5' | GCATGGGTGGTTCAGTGG | 351.39 | 4.72E-90 | 3.52 | ** |
| hsa-miR-582-5p | TTACAGTTGTTCAACCAGTTACT | 5' | TACAGTTGTTCAACCAGTTAC | 1.1561 | 5' | TTACAGTTGTTCAACCAGTTAC | 4.07 | 0.040367721 | 3.52 |  |
| hsa-miR-486-5p | TCCTGTACTGAGCTGCCCCGAG | 5' | TCCTGTACTGAGCTGCCCCGAG | 6.5513 | 5' | TCCTGTACTGAGCTGCCCCGAG | 23.04 | 3.34E-07 | 3.52 | ** |
| hsa-let-7d* | CTATACGACCTGCTGCCTTTCT | 3' | CTATACGACCTGCTGCCTT | 3.083 | 3' | CTATACGACCTGCTGCCTTTCT | 10.84 | 0.000534949 | 3.52 | ** |
| hsa-let-7a* | CTATACAATCTACTGTCTTTC | 3' | ATACAATCTACTGTCTTT | 3.8537 | 3' | ATACAATCTACTGTCTTTCCT | 13.22 | 0.000154384 | 3.43 | ** |
| hsa-miR-197 | TTCACCACCTTCTCCACCCAGC | 3' | TTCACCACCTTCTCCACCCAGC | 1.1561 | 3' | TTCACCACCTTCTCCACCCAGC | 3.73 | 0.06284105 | 3.22 |  |
| hsa-miR-542-3p | TGTGACAGATTGATAACTGAAA | 3' | TTGTGACAGATTGATAAC | 1.1561 | 3' | TGTGACAGATTGATAACT | 3.73 | 0.06284105 | 3.22 |  |
| hsa-miR-146b-5p | TGAGAACTGAATTCCATAGGCT | 5' | TGAGAACTGAATTCCATAGGCT | 5.3952 | 5' | TGAGAACTGAATTCCATAGGCT | 15.25 | 0.000308409 | 2.83 | ** |
| hsa-miR-1301 | TTGCAGCTGCCTGGGAGTGACTTC | 3' | TTGCAGCTGCCTGGGAGTGACT | 6.9367 | 3' | TTGCAGCTGCCTGGGAGTGACT | 19.31 | 5.59E-05 | 2.78 | ** |
| hsa-miR-744 | TGCGGGGCTAGGGCTAACAGCA | 5' | TGCGGGGCTAGGGCTAACAGCA | 10.7905 | 5' | TGCGGGGCTAGGGCTAACAGCA | 27.79 | 4.89E-06 | 2.58 | ** |
| hsa-miR-125b-2* | TCACAAGTCAGGCTCTTGGGAC | 3' | TCACAAGTCAGGCTCTTG | 43.9325 | 3' | ACAAGTCAGGCTCTTGGG | 112.50 | 3.39E-20 | 2.56 | ** |
| hsa-miR-27a | TTCACAGTGGCTAAGTTCCGC | 3' | TTCACAGTGGCTAAGTTC | 38.9227 | 3' | TTCACAGTGGCTAAGTTC | 98.95 | 9.49E-18 | 2.54 | ** |
| hsa-miR-320c | AAAAGCTGGGTTGAGAGGGT | 3' | AAAAGCTGGGTTGAGAGGG | 124.0902 | 3' | AAAAGCTGGGTTGAGAGGG | 301.92 | 2.05E-47 | 2.43 | ** |
| hsa-miR-27b | TTCACAGTGGCTAAGTTCTGC | 3' | TTCACAGTGGCTAAGTTC | 43.1618 | 3' | TTCACAGTGGCTAAGTTC | 103.35 | 7.88E-17 | 2.39 | ** |
| hsa-miR-320a | AAAAGCTGGGTTGAGAGGGCGA | 3' | AAAAGCTGGGTTGAGAGGGCGA | 4903.1037 | 3' | AAAAGCTGGGTTGAGAGGGCGA | 11647.81 | 0 | 2.38 | ** |
| hsa-miR-1272 | GATGATGATGGCAGCAAATTCTGAAA | 5' | GATGATGATGGCAGCAAATT | 7.7075 | 5' | GATGATGATGGCAGCAAATT | 18.30 | 0.00056716 | 2.37 | ** |
| hsa-miR-127-3p | TCGGATCCGTCTGAGCTTGGCT | 3' | TCGGATCCGTCTGAGCTTGGCT | 1 | 3' | TCGGATCCGTCTGAGCTTGGCT | 2.37 | 0.162284235 | 2.37 |  |
| hsa-miR-183* | GTGAATTACCGAAGGGCCATAA | 3' | TGAATTACCGAAGGGCCATA | 5.3952 | 3' | TGAATTACCGAAGGGCCATAA | 12.54 | 0.005382147 | 2.32 | ** |
| hsa-miR-1259 | ATATATGATGACTTAGCTTTT | 5' | ATATATGATGACTTAGCTTTTTT | 17.7272 | 5' | ATATATGATGACTTAGCTTTTTT | 41.00 | 4.00E-07 | 2.31 | ** |
| hsa-miR-767-5p | TGCACCATGGTTGTCTGAGCATG | 5' | ATGCACCATGGTTGTCTGA | 1.9269 | 5' | TGCACCATGGTTGTCTGA | 4.41 | 0.11499865 | 2.29 |  |
| hsa-miR-320d | AAAAGCTGGGTTGAGAGGA | 3' | AAAAGCTGGGTTGAGAGG | 33.9128 | 3' | AAAAGCTGGGTTGAGAGG | 77.26 | 6.17E-12 | 2.28 | ** |
| hsa-miR-423-5p | TGAGGGGCAGAGAGCGAGACTTT | 5' | TGAGGGGCAGAGAGCGAGACTTT | 1838.6157 | 5' | TGAGGGGCAGAGAGCGAGACTTT | 4158.43 | 0 | 2.26 | ** |
| hsa-miR-1287 | TGCTGGATCAGTGGTTCGAGTC | 5' | TGCTGGATCAGTGGTTCGAGTC | 1.1561 | 5' | TGCTGGATCAGTGGTTCGAGTC | 2.37 | 0.320290675 | 2.05 |  |
| hsa-miR-345 | GCTGACTCCTAGTCCAGGGCTC | 5' | GCTGACTCCTAGTCCAGGGCT | 1.1561 | 5' | GCTGACTCCTAGTCCAGG | 2.37 | 0.320290675 | 2.05 |  |
| hsa-miR-411 | TAGTAGACCGTATAGCGTACG | 5' | TAGTAGACCGTATAGCGTAC | 1.1561 | 5' | TAGTAGACCGTATAGCGTAC | 2.37 | 0.320290675 | 2.05 |  |
| hsa-miR-548d-5p | AAAAGTAATTGTGGTTTTTGCC | 5' | AAAAGTAATTGTGGTTTTT | 1.1561 | 5' | AAAAGTAATTGTGGTTTTT | 2.37 | 0.320290675 | 2.05 |  |
| hsa-miR-103-2* | AGCTTCTTTACAGTGCTGCCTTG | 5' | AGCTTCTTTACAGTGCTGCCTTGTA | 1 | 5' | AGCTTCTTTACAGTGCTGCCTTGT | 2.03 | 0.251366258 | 2.03 |  |
| hsa-miR-320b | AAAAGCTGGGTTGAGAGGGCAA | 3' | AAAAGCTGGGTTGAGAGGGC | 929.1349 | 3' | AAAAGCTGGGTTGAGAGGGC | 1877.60 | 7.25E-197 | 2.02 | ** |
| hsa-miR-199a-3p | ACAGTAGTCTGCACATTGGTTA | 3' | ACAGTAGTCTGCACATTGGTT | 134.8806 | 3' | ACAGTAGTCTGCACATTGGTT | 270.41 | 2.41E-29 | 2.00 | ** |
| hsa-miR-199b-3p | ACAGTAGTCTGCACATTGGTTA | 3' | ACAGTAGTCTGCACATTGGTT | 134.8806 | 3' | ACAGTAGTCTGCACATTGGTT | 270.41 | 2.41E-29 | 2.00 | ** |
| hsa-miR-30e | TGTAAACATCCTTGACTGGAAG | 5' | TGTAAACATCCTTGACTGGAAGC | 29.6737 | 5' | TGTAAACATCCTTGACTGGAAGC | 56.59 | 1.42E-06 | 1.91 |  |
| hsa-miR-25 | CATTGCACTTGTCTCGGTCTGA | 3' | CATTGCACTTGTCTCGGTCT | 411.964 | 3' | CATTGCACTTGTCTCGGTCT | 768.18 | 4.24E-67 | 1.86 |  |
| hsa-miR-183 | TATGGCACTGGTAGAATTCACT | 5' | TATGGCACTGGTAGAATTCACT | 11.9466 | 5' | TATGGCACTGGTAGAATTCACT | 22.03 | 0.00424865 | 1.84 |  |
| hsa-miR-182 | TTTGGCAATGGTAGAACTCACACT | 5' | TTTGGCAATGGTAGAACTCACACT | 9.249 | 5' | TTTGGCAATGGTAGAACTCACACTG | 16.94 | 0.013202453 | 1.83 |  |
| hsa-miR-451 | AAACCGTTACCATTACTGAGTT | 5' | AAACCGTTACCATTACTGAGTT | 18.4979 | 5' | AAACCGTTACCATTACTGAGTT | 33.21 | 0.000695586 | 1.80 |  |
| hsa-miR-99b | CACCCGTAGAACCGACCTTGCG | 5' | CACCCGTAGAACCGACCTTGCG | 106.7484 | 5' | CACCCGTAGAACCGACCTTGCG | 190.10 | 7.61E-16 | 1.78 |  |
| hsa-miR-1978 | GGTTTGGTCCTAGCCTTTCTA | 3' | ATAGGTTTGGTCCTAGCCTTTCT | 1.1561 | 3' | ATAGGTTTGGTCCTAGCCTTTCT | 2.03 | 0.45922431 | 1.76 |  |
| hsa-miR-99b* | CAAGCTCGTGTCTGTGGGTCCG | 3' | CAAGCTCGTGTCTGTGGGTCC | 5.0099 | 3' | CAAGCTCGTGTCTGTGGGTCC | 8.81 | 0.09593359 | 1.76 |  |
| hsa-miR-23b* | TGGGTTCCTGGCATGCTGATTT | 5' | GGGTTCCTGGCATGCTGATTT | 26.5908 | 5' | GGGTTCCTGGCATGCTGATTT | 45.75 | 0.00018612 | 1.72 |  |
| hsa-miR-92b* | AGGGACGGGACGCGGTGCAGTG | 5' | AGGGACGGGACGCGGTGCAGTGT | 105.2069 | 5' | AGGGACGGGACGCGGTGCAGTGT | 178.92 | 3.35E-13 | 1.70 |  |
| hsa-miR-143* | AGGGACGGGACGCGGTGCAGTG | 5' | AGGGACGGGACGCGGTGCAGTGT | 1 | 5' | AGGGACGGGACGCGGTGCAGTGT | 1.69 | 0.045401044 | 1.69 |  |
| hsa-miR-144* | AGGGACGGGACGCGGTGCAGTG | 5' | AGGGACGGGACGCGGTGCAGTGT | 1 | 5' | AGGGACGGGACGCGGTGCAGTGT | 1.69 | 0.045401044 | 1.69 |  |
| hsa-miR-449c | TAGGCAGTGTATTGCTAGCGGCTGT | 5' | AGGCAGTGTATTGCTAGC | 1 | 5' | AGGCAGTGTATTGCTAGC | 1.69 | 0.381574845 | 1.69 |  |
| hsa-miR-491-5p | AGTGGGGAACCCTTCCATGAGG | 5' | AGTGGGGAACCCTTCCATGAG | 1 | 5' | AGTGGGGAACCCTTCCATGAG | 1.69 | 0.172855691 | 1.69 |  |
| hsa-miR-518e* | CTCTAGAGGGAAGCGCTTTCTG | 5' | CTCTAGAGGGAAGCGCTTTC | 1 | 5' | CTCTAGAGGGAAGCGCTTTCT | 1.69 | 0.381574845 | 1.69 |  |
| hsa-miR-519a* | CTCTAGAGGGAAGCGCTTTCTG | 5' | CTCTAGAGGGAAGCGCTTTC | 1 | 5' | CTCTAGAGGGAAGCGCTTTCT | 1.69 | 0.381574845 | 1.69 |  |
| hsa-miR-519b-5p | CTCTAGAGGGAAGCGCTTTCTG | 5' | CTCTAGAGGGAAGCGCTTTC | 1 | 5' | CTCTAGAGGGAAGCGCTTTCT | 1.69 | 0.381574845 | 1.69 |  |
| hsa-miR-519c-5p | CTCTAGAGGGAAGCGCTTTCTG | 5' | CTCTAGAGGGAAGCGCTTTC | 1 | 5' | CTCTAGAGGGAAGCGCTTTCT | 1.69 | 0.381574845 | 1.69 |  |
| hsa-miR-522* | CTCTAGAGGGAAGCGCTTTCTG | 5' | CTCTAGAGGGAAGCGCTTTC | 1 | 5' | CTCTAGAGGGAAGCGCTTTCT | 1.69 | 0.381574845 | 1.69 |  |
| hsa-miR-523* | CTCTAGAGGGAAGCGCTTTCTG | 5' | CTCTAGAGGGAAGCGCTTTC | 1 | 5' | CTCTAGAGGGAAGCGCTTTCT | 1.69 | 0.381574845 | 1.69 |  |
| hsa-miR-185 | TGGAGAGAAAGGCAGTTCCTGA | 5' | TGGAGAGAAAGGCAGTTCCT | 2415.5194 | 5' | TGGAGAGAAAGGCAGTTCCT | 4081.85 | 4.59E-260 | 1.69 |  |
| hsa-miR-7-1* | CAACAAATCACAGTCTGCCATA | 3' | CAACAAATCACAGTCTGCCATA | 7.3221 | 3' | CAACAAATCACAGTCTGCCATA | 12.20 | 0.070690029 | 1.67 |  |
| hsa-miR-499-5p | TTAAGACTTGCAGTGATGTTT | 5' | TTAAGACTTGCAGTGATGTTTA | 55.1084 | 5' | TTAAGACTTGCAGTGATGTTTA | 88.44 | 3.81E-06 | 1.60 |  |
| hsa-miR-330-3p | GCAAAGCACACGGCCTGCAGAGA | 3' | GCAAAGCACACGGCCTGCAGAGA | 97.4994 | 3' | GCAAAGCACACGGCCTGCAGAGA | 156.21 | 8.81E-10 | 1.60 |  |
| hsa-miR-148a* | AAAGTTCTGAGACACTCCGACT | 5' | AAAGTTCTGAGACACTCC | 1.9269 | 5' | AAAGTTCTGAGACACTCCGA | 3.05 | 0.434639824 | 1.58 |  |
| hsa-miR-181a* | ACCATCGACCGTTGATTGTACC | 3' | ACCATCGACCGTTGATTGTAC | 1.9269 | 3' | ACCATCGACCGTTGATTG | 3.05 | 0.434639824 | 1.58 |  |
| hsa-miR-1255a | AGGATGAGCAAAGAAAGTAGATT | 5' | AGGATGAGCAAAGAAAGT | 54.3376 | 5' | AGGATGAGCAAAGAAAGT | 85.05 | 1.56E-05 | 1.57 |  |
| hsa-miR-17* | ACTGCAGTGAAGGCACTTGTAG | 3' | ACTGCAGTGAAGGCACTTG | 267.0637 | 3' | ACTGCAGTGAAGGCACTTG | 417.81 | 9.34E-22 | 1.56 |  |
| hsa-let-7c | TGAGGTAGTAGGTTGTATGGTT | 5' | TGAGGTAGTAGGTTGTATGGTT | 172812.9202 | 5' | TGAGGTAGTAGGTTGTATGGTT | 266022.34 | 0 | 1.54 |  |
| hsa-miR-219-5p | TGATTGTCCAAACGCAATTCT | 5' | TGATTGTCCAAACGCAAT | 1.5415 | 5' | TGATTGTCCAAACGCAAT | 2.37 | 0.523594743 | 1.54 |  |
| hsa-miR-199b-5p | CCCAGTGTTTAGACTATCTGTTC | 5' | CCCAGTGTTTAGACTATCT | 7.3221 | 5' | CCCAGTGTTTAGACTATCT | 11.18 | 0.143065163 | 1.53 |  |
| hsa-miR-1306 | ACGTTGGCTCTGGTGGTG | 3' | ACGTTGGCTCTGGTGGTGAT | 4.2391 | 3' | ACGTTGGCTCTGGTGGTGAT | 6.44 | 0.279030481 | 1.52 |  |
| hsa-miR-424* | CAAAACGTGAGGCGCTGCTAT | 3' | CAAAACGTGAGGCGCTGCTAT | 25.82 | 3' | CAAAACGTGAGGCGCTGCTATA | 38.97 | 0.006876092 | 1.51 |  |
| hsa-let-7e | TGAGGTAGGAGGTTGTATAGTT | 5' | TGAGGTAGGAGGTTGTATAGTT | 10055.5438 | 5' | TGAGGTAGGAGGTTGTATAGTT | 15002.14 | 0 | 1.49 |  |
| hsa-miR-503 | TAGCAGCGGGAACAGTTCTGCAG | 5' | TAGCAGCGGGAACAGTTC | 78.6161 | 5' | TAGCAGCGGGAACAGTTC | 116.91 | 5.44E-06 | 1.49 |  |
| hsa-miR-1303 | TTTAGAGACGGGGTCTTGCTCT | 3' | TTTTAGAGACGGGGTCTTGCT | 8.4782 | 3' | TTTTAGAGACGGGGTCTTGCT | 12.54 | 0.147846871 | 1.48 |  |
| hsa-miR-2110 | TTGGGGAAACGGCCGCTGAGTG | 5' | TTGGGGAAACGGCCGCTGAGTGA | 14.2588 | 5' | TTGGGGAAACGGCCGCTGAGTGA | 21.01 | 0.061365523 | 1.47 |  |
| hsa-miR-371-5p | ACTCAAACTGTGGGGGCACT | 5' | ACTCAAACTGTGGGGGCAC | 1.1561 | 5' | ACTCAAACTGTGGGGGCAC | 1.69 | 0.641992019 | 1.47 |  |
| hsa-miR-574-5p | TGAGTGTGTGTGTGTGAGTGTGT | 5' | TGAGTGTGTGTGTGTGAGTGTGTGT | 1.1561 | 5' | TGAGTGTGTGTGTGTGAGT | 1.69 | 0.641992019 | 1.47 |  |
| hsa-miR-374b* | CTTAGCAGGTTGTATTATCATT | 3' | CTTAGCAGGTTGTATTATCATT | 4.6245 | 3' | CTTAGCAGGTTGTATTATCATT | 6.78 | 0.304897234 | 1.47 |  |
| hsa-miR-30d | TGTAAACATCCCCGACTGGAAG | 5' | TGTAAACATCCCCGACTGGAAGC | 137.1929 | 5' | TGTAAACATCCCCGACTGGAAGCT | 197.89 | 3.62E-08 | 1.44 |  |
| hsa-miR-125b | TCCCTGAGACCCTAACTTGTGA | 5' | TCCCTGAGACCCTAACTTGTG | 347.992 | 5' | TCCCTGAGACCCTAACTTGTGA | 500.15 | 3.78E-18 | 1.44 |  |
| hsa-miR-660 | TACCCATTGCATATCGGAGTTG | 5' | TACCCATTGCATATCGGAGTT | 9.6343 | 5' | TACCCATTGCATATCGGAGTT | 13.55 | 0.183312465 | 1.41 |  |
| hsa-miR-149* | AGGGAGGGACGGGGGCTGTGC | 3' | GAGGGAGGGACGGGGGCTGTGC | 1.9269 | 3' | GAGGGAGGGACGGGGGCTGTGC | 2.71 | 0.578314209 | 1.41 |  |
| hsa-miR-1908 | CGGCGGGGACGGCGATTGGTC | 5' | CGGCGGGGACGGCGATTGGT | 1.9269 | 5' | CGGCGGGGACGGCGATTGGTC | 2.71 | 0.578314209 | 1.41 |  |
| hsa-miR-33a | GTGCATTGTAGTTGCATTGCA | 5' | GTGCATTGTAGTTGCATT | 154.1493 | 5' | GTGCATTGTAGTTGCATT | 213.14 | 3.19E-07 | 1.38 |  |
| hsa-let-7d | AGAGGTAGTAGGTTGCATAGTT | 5' | AGAGGTAGTAGGTTGCATAGTT | 73632.8788 | 5' | AGAGGTAGTAGGTTGCATAGTT | 100277.76 | 0 | 1.36 |  |
| hsa-miR-1248 | ACCTTCTTGTATAAGCACTGTGCTAAA | 5' | ACCTTCTTGTATAAGCACT | 1 | 5' | ACCTTCTTGTATAAGCACTGTGCT | 1.36 | 0.284925411 | 1.36 |  |
| hsa-miR-1256 | ACCTTCTTGTATAAGCACTGTGCTAAA | 5' | ACCTTCTTGTATAAGCACT | 1 | 5' | ACCTTCTTGTATAAGCACTGTGCT | 1.36 | 0.085321772 | 1.36 |  |
| hsa-miR-137 | ACCTTCTTGTATAAGCACTGTGCTAAA | 5' | ACCTTCTTGTATAAGCACT | 1 | 5' | ACCTTCTTGTATAAGCACTGTGCT | 1.36 | 0.085321772 | 1.36 |  |
| hsa-miR-185* | AGGGGCTGGCTTTCCTCTGGTC | 3' | CAGGGGCTGGCTTTCCTC | 1 | 3' | CAGGGGCTGGCTTTCCTC | 1.36 | 0.56509971 | 1.36 |  |
| hsa-miR-217 | TACTGCATCAGGAACTGATTGGA | 5' | TACTGCATCAGGAACTGATTGG | 1 | 5' | TACTGCATCAGGAACTGATTGG | 1.36 | 0.284925411 | 1.36 |  |
| hsa-miR-369-3p | AATAATACATGGTTGATCTTT | 3' | AATAATACATGGTTGATCTTT | 1 | 3' | AATAATACATGGTTGATCTTT | 1.36 | 0.56509971 | 1.36 |  |
| hsa-miR-450a | TTTTGCGATGTGTTCCTAATAT | 5' | TTTTGCGATGTGTTCCTAATAT | 1 | 5' | TTTTGCGATGTGTTCCTAATAT | 1.36 | 0.56509971 | 1.36 |  |
| hsa-miR-495 | AAACAAACATGGTGCACTTCTT | 3' | AAACAAACATGGTGCACTTCTTT | 1 | 3' | AAACAAACATGGTGCACTTCT | 1.36 | 0.284925411 | 1.36 |  |
| hsa-miR-720 | TCTCGCTGGGGCCTCCA | 5' | TCTCGCTGGGGCCTCCAAA | 1 | 5' | ATCTCGCTGGGGCCTCCA | 1.36 | 0.284925411 | 1.36 |  |
| hsa-miR-548e | AAAAACTGAGACTACTTTTGCA | 3' | AAAAACTGAGACTACTTTTGCA | 2.3122 | 3' | AAAAACTGAGACTACTTTTGCA | 3.05 | 0.62576827 | 1.32 |  |
| hsa-miR-1254 | AGCCTGGAAGCTGGAGCCTGCAGT | 5' | GAGCCTGGAAGCTGGAGCCTGCA | 5.3952 | 5' | AGCCTGGAAGCTGGAGCCTGCAGTG | 7.12 | 0.43497956 | 1.32 |  |
| hsa-miR-30e* | CTTTCAGTCGGATGTTTACAGC | 3' | CTTTCAGTCGGATGTTTACAGC | 20.8102 | 3' | CTTTCAGTCGGATGTTTACAG | 26.77 | 0.155061719 | 1.29 |  |
| hsa-miR-151-5p | TCGAGGAGCTCACAGTCTAGT | 5' | TCGAGGAGCTCACAGTCTAGTA | 26.2054 | 5' | TCGAGGAGCTCACAGTCTAGTA | 32.87 | 0.153087764 | 1.25 |  |
| hsa-miR-92b | TATTGCACTCGTCCCGGCCTCC | 3' | TATTGCACTCGTCCCGGCC | 288.2592 | 3' | TATTGCACTCGTCCCGGCC | 351.73 | 3.11E-05 | 1.22 |  |
| hsa-miR-365* | AGGGACTTTCAGGGGCAGCTGT | 5' | AGGGACTTTCAGGGGCAGCT | 106.363 | 5' | GAGGGACTTTCAGGGGCAGCT | 129.10 | 0.014027747 | 1.21 |  |
| hsa-miR-148b* | AAGTTCTGTTATACACTCAGGC | 5' | GAAGTTCTGTTATACACTCAGGCT | 4.2391 | 5' | GAAGTTCTGTTATACACTCAGGCT | 5.08 | 0.665476182 | 1.20 |  |
| hsa-miR-10a | TACCCTGTAGATCCGAATTTGTG | 5' | TACCCTGTAGATCCGAATTTG | 5.3952 | 5' | TACCCTGTAGATCCGAATTTG | 6.44 | 0.631134569 | 1.19 |  |
| hsa-miR-99a | AACCCGTAGATCCGATCTTGTG | 5' | AACCCGTAGATCCGATCTTGT | 1579.2595 | 5' | AACCCGTAGATCCGATCTTGT | 1882.00 | 1.26E-17 | 1.19 |  |
| hsa-miR-1292 | TGGGAACGGGTTCCGGCAGACGCTG | 5' | GGGAACGGGTTCCGGCAGACGC | 11.9466 | 5' | GGGAACGGGTTCCGGCAGACGC | 14.23 | 0.467777303 | 1.19 |  |
| hsa-miR-625 | AGGGGGAAAGTTCTATAGTCC | 5' | AGGGGGAAAGTTCTATAGTC | 1.1561 | 5' | AGGGGGAAAGTTCTATAGTC | 1.36 | 0.870974487 | 1.17 |  |
| hsa-miR-765 | TGGAGGAGAAGGAAGGTGATG | 3' | TCTGGAGGAGAAGGAAGGTGATG | 2.3122 | 3' | TGGAGGAGAAGGAAGGTGATG | 2.71 | 0.793825785 | 1.17 |  |
| hsa-let-7b | TGAGGTAGTAGGTTGTGTGGTT | 5' | TGAGGTAGTAGGTTGTGTG | 66604.0564 | 5' | TGAGGTAGTAGGTTGTGTG | 77058.05 | 0 | 1.16 |  |
| hsa-miR-340 | TTATAAAGCAATGAGACTGATT | 5' | TTATAAAGCAATGAGACTGATT | 143.7442 | 5' | TTATAAAGCAATGAGACTGATT | 164.34 | 0.051722108 | 1.14 |  |
| hsa-miR-342-5p | AGGGGTGCTATCTGTGATTGA | 5' | AGGGGTGCTATCTGTGATTGA | 2.6976 | 5' | GGGGTGCTATCTGTGATTGAGGG | 3.05 | 0.830170606 | 1.13 |  |
| hsa-miR-148a | TCAGTGCACTACAGAACTTTGT | 3' | TCAGTGCACTACAGAACT | 135.266 | 3' | TCAGTGCACTACAGAACT | 152.82 | 0.086502981 | 1.13 |  |
| hsa-let-7g | TGAGGTAGTAGTTTGTACAGTT | 5' | TGAGGTAGTAGTTTGTACAGTT | 1854.416 | 5' | TGAGGTAGTAGTTTGTACAGTT | 2063.29 | 2.98E-08 | 1.11 |  |
| hsa-miR-374a | TTATAATACAACCTGATAAGTG | 5' | TTATAATACAACCTGATAA | 10.4051 | 5' | TTATAATACAACCTGATAAGTG | 11.52 | 0.703025732 | 1.11 |  |
| hsa-miR-24 | TGGCTCAGTTCAGCAGGAACAG | 3' | TGGCTCAGTTCAGCAGGAACAG | 64.3573 | 3' | TGGCTCAGTTCAGCAGGAACAG | 71.16 | 0.334651724 | 1.11 |  |
| hsa-miR-99a* | CAAGCTCGCTTCTATGGGTCTG | 3' | CAAGCTCGCTTCTATGGG | 9.6343 | 3' | CAAGCTCGCTTCTATGGG | 10.17 | 0.85337682 | 1.06 |  |
| hsa-miR-421 | ATCAACAGACATTAATTGGGCGC | 3' | ATCAACAGACATTAATTG | 15.8003 | 3' | ATCAACAGACATTAATTG | 16.60 | 0.822739483 | 1.05 |  |
| hsa-miR-34a* | CAATCAGCAAGTATACTGCCCT | 3' | AATCAGCAAGTATACTGCCCTA | 16.1857 | 3' | AATCAGCAAGTATACTGCCCTA | 16.94 | 0.835032192 | 1.05 |  |
| hsa-miR-192 | CTGACCTATGAATTGACAGCC | 5' | CTGACCTATGAATTGACAGCC | 237.0045 | 5' | CTGACCTATGAATTGACAGCC | 244.31 | 0.581606583 | 1.03 |  |
| hsa-miR-1228* | CTGACCTATGAATTGACAGCC | 5' | CTGACCTATGAATTGACAGCC | 1 | 5' | CTGACCTATGAATTGACAGCC | 1.02 | 0.460435088 | 1.02 |  |
| hsa-miR-1299 | CTGACCTATGAATTGACAGCC | 5' | CTGACCTATGAATTGACAGCC | 1 | 5' | CTGACCTATGAATTGACAGCC | 1.02 | 0.160344435 | 1.02 |  |
| hsa-miR-152 | TCAGTGCATGACAGAACTTGG | 3' | TCAGTGCATGACAGAACTTGG | 1 | 3' | TCAGTGCATGACAGAACT | 1.02 | 0.811454441 | 1.02 |  |
| hsa-miR-625* | TCAGTGCATGACAGAACTTGG | 3' | TCAGTGCATGACAGAACTTGG | 1 | 3' | TCAGTGCATGACAGAACT | 1.02 | 0.160344435 | 1.02 |  |
| hsa-miR-342-3p | TCTCACACAGAAATCGCACCCGT | 3' | TCTCACACAGAAATCGCACCCGTC | 387.3001 | 3' | TCTCACACAGAAATCGCACCCGTC | 391.38 | 0.809625861 | 1.01 |  |
| hsa-miR-103 | AGCAGCATTGTACAGGGCTATGA | 3' | AGCAGCATTGTACAGGGCTAT | 14157.8419 | 3' | AGCAGCATTGTACAGGGCTATG | 14116.03 | 0.679216727 | 1.00 |  |
| hsa-miR-26b | TTCAAGTAATTCAGGATAGGT | 5' | TTCAAGTAATTCAGGATAGGTT | 245.8681 | 5' | TTCAAGTAATTCAGGATAGGTT | 243.30 | 0.845107925 | 1.01 |  |
| hsa-miR-1307 | ACTCGGCGTGGCGTCGGTCGTG | 3' | CTCGGCGTGGCGTCGGTCGTG | 240.8583 | 3' | CTCGGCGTGGCGTCGGTCGTG | 238.22 | 0.839175243 | 1.01 |  |
| hsa-miR-151-3p | CTAGACTGAAGCTCCTTGAGG | 3' | CTAGACTGAAGCTCCTTGAGGA | 24.6639 | 3' | CTAGACTGAAGCTCCTTGAGGA | 24.06 | 0.880106155 | 1.03 |  |
| hsa-miR-128 | TCACAGTGAACCGGTCTCTTT | 3' | TCACAGTGAACCGGTCTCTTT | 2578.1469 | 3' | TCACAGTGAACCGGTCTCTTT | 2506.17 | 0.093282443 | 1.03 |  |
| hsa-miR-92a | TATTGCACTTGTCCCGGCCTGT | 3' | TATTGCACTTGTCCCGGCCT | 6984.5045 | 3' | TATTGCACTTGTCCCGGCCT | 6711.37 | 0.000104521 | 1.04 |  |
| hsa-miR-193a-5p | TGGGTCTTTGCGGGCGAGATGA | 5' | TGGGTCTTTGCGGGCGAGATGA | 35.069 | 5' | TGGGTCTTTGCGGGCGAGATGA | 32.87 | 0.653710916 | 1.07 |  |
| hsa-miR-9 | TCTTTGGTTATCTAGCTGTATGA | 5' | TCTTTGGTTATCTAGCTGTATG | 20.4248 | 5' | TCTTTGGTTATCTAGCTGTATG | 18.98 | 0.69659832 | 1.08 |  |
| hsa-miR-107 | AGCAGCATTGTACAGGGCTATCA | 3' | AGCAGCATTGTACAGGGCTAT | 10093.6958 | 3' | AGCAGCATTGTACAGGGCTAT | 9360.20 | 2.25E-18 | 1.08 |  |
| hsa-miR-1826 | AGCAGCATTGTACAGGGCTATCA | 3' | AGCAGCATTGTACAGGGCTAT | 8.8636 | 3' | AGCAGCATTGTACAGGGCTAT | 8.13 | 0.761399716 | 1.09 |  |
| hsa-miR-1974 | TGGTTGTAGTCCGTGCGAGAATA | 3' | GTGGTTGTAGTCCGTGCGAGAA | 102.8947 | 3' | GTGGTTGTAGTCCGTGCGAGAAT | 92.17 | 0.200681084 | 1.12 |  |
| hsa-miR-574-3p | CACGCTCATGCACACACCCACA | 3' | CACGCTCATGCACACACCCACA | 23.8931 | 3' | CACGCTCATGCACACACCCACA | 21.35 | 0.52600914 | 1.12 |  |
| hsa-miR-19a* | AGTTTTGCATAGTTGCACTACA | 5' | AGTTTTGCATAGTTGCACTA | 1.1561 | 5' | AGTTTTGCATAGTTGCACTA | 1.02 | 0.860072585 | 1.14 |  |
| hsa-miR-548j | AAAAGTAATTGCGGTCTTTGGT | 5' | AAAAGTAATTGCGGTCTTT | 1.1561 | 5' | AAAAGTAATTGCGGTCTTT | 1.02 | 0.860072585 | 1.14 |  |
| hsa-miR-18a* | ACTGCCCTAAGTGCTCCTTCTGG | 3' | ACTGCCCTAAGTGCTCCTTCT | 2.6976 | 3' | ACTGCCCTAAGTGCTCCTTCT | 2.37 | 0.800108395 | 1.14 |  |
| hsa-miR-1291 | TGGCCCTGACTGAAGACCAGCAGT | 5' | GTGGCCCTGACTGAAGACCAGCA | 13.8734 | 5' | GTGGCCCTGACTGAAGACCAGCA | 12.20 | 0.58146661 | 1.14 |  |
| hsa-miR-28-3p | CACTAGATTGTGAGCTCCTGGA | 3' | CACTAGATTGTGAGCTCCTGGA | 17.3418 | 3' | CACTAGATTGTGAGCTCCTGGA | 15.25 | 0.538523355 | 1.14 |  |
| hsa-miR-106b* | CCGCACTGTGGGTACTTGCTGC | 3' | CCGCACTGTGGGTACTTGCTGC | 10.7905 | 3' | CCGCACTGTGGGTACTTGCTGC | 9.49 | 0.625854241 | 1.14 |  |
| hsa-miR-32* | CAATTTAGTGTGTGTGATATTT | 3' | AATTTAGTGTGTGTGATATTT | 1.5415 | 3' | CAATTTAGTGTGTGTGATATT | 1.36 | 0.842797427 | 1.14 |  |
| hsa-miR-125a-5p | TCCCTGAGACCCTTTAACCTGTGA | 5' | TCCCTGAGACCCTTTAACCT | 143.7442 | 5' | TCCCTGAGACCCTTTAACCT | 126.05 | 0.072913968 | 1.14 |  |
| hsa-miR-18b | TAAGGTGCATCTAGTGCAGTTAG | 5' | TAAGGTGCATCTAGTGCA | 1.1561 | 5' | TAAGGTGCATCTAGTGCA | 1.00 | 0.571249267 | 1.16 |  |
| hsa-miR-1977 | GATTAGGGTGCTTAGCTGTTAA | 5' | GATTAGGGTGCTTAGCTGTTAACT | 1.1561 | 5' | TAGGGTGCTTAGCTGTTA | 1.00 | 0.29985774 | 1.16 |  |
| hsa-miR-212 | TAACAGTCTCCAGTCACGGCC | 3' | TAACAGTCTCCAGTCACGGCCA | 1.1561 | 5' | TAGGGTGCTTAGCTGTTA | 1.00 | 0.095848283 | 1.16 |  |
| hsa-miR-361-3p | TCCCCCAGGTGTGATTCTGATTT | 3' | CCCCCAGGTGTGATTCTGATTTGC | 1.1561 | 3' | TCCCCCAGGTGTGATTCT | 1.00 | 0.29985774 | 1.16 |  |
| hsa-miR-485-5p | AGAGGCTGGCCGTGATGAATTC | 5' | AGAGGCTGGCCGTGATGAATT | 1.1561 | 5' | AGAGGCTGGCCGTGATGAATT | 1.00 | 0.29985774 | 1.16 |  |
| hsa-miR-501-5p | AATCCTTTGTCCCTGGGTGAGA | 5' | AATCCTTTGTCCCTGGGTGAGA | 1.1561 | 5' | AGAGGCTGGCCGTGATGAATT | 1.00 | 0.095848283 | 1.16 |  |
| hsa-miR-548a-3p | CAAAACTGGCAATTACTTTTGC | 3' | CAAAACTGGCAATTACTTTT | 1.1561 | 5' | AGAGGCTGGCCGTGATGAATT | 1.00 | 0.095848283 | 1.16 |  |
| hsa-miR-95 | TTCAACGGGTATTTATTGAGCA | 3' | TTCAACGGGTATTTATTG | 1.1561 | 3' | TTCAACGGGTATTTATTG | 1.00 | 0.29985774 | 1.16 |  |
| hsa-let-7f | TGAGGTAGTAGATTGTATAGTT | 5' | TGAGGTAGTAGATTGTATAGTT | 276185.437 | 5' | TGAGGTAGTAGATTGTATAGTT | 236017.39 | 0 | 1.17 |  |
| hsa-miR-339-3p | TGAGCGCCTCGACGACAGAGCCG | 3' | AGCGCCTCGACGACAGAG | 12.7173 | 3' | TGAGCGCCTCGACGACAG | 10.84 | 0.517433866 | 1.17 |  |
| hsa-miR-18a | TAAGGTGCATCTAGTGCAGATAG | 5' | TAAGGTGCATCTAGTGCAGAT | 12.3319 | 5' | TAAGGTGCATCTAGTGCAGATA | 10.50 | 0.521351926 | 1.17 |  |
| hsa-miR-335 | TCAAGAGCAATAACGAAAAATGT | 5' | TCAAGAGCAATAACGAAAAATG | 18.8833 | 5' | TCAAGAGCAATAACGAAAAATGT | 15.93 | 0.402121163 | 1.19 |  |
| hsa-miR-30b* | CTGGGAGGTGGATGTTTACTTC | 3' | CTGGGAGGTGGATGTTTACT | 34.2982 | 3' | CTGGGAGGTGGATGTTTACT | 28.80 | 0.24845932 | 1.19 |  |
| hsa-miR-1255b | CGGATGAGCAAAGAAAGTGGTT | 5' | CGGATGAGCAAAGAAAGTGGTT | 7.7075 | 5' | CGGATGAGCAAAGAAAGTGGTT | 6.44 | 0.570702216 | 1.20 |  |
| hsa-miR-210 | CTGTGCGTGTGACAGCGGCTGA | 3' | CTGTGCGTGTGACAGCGGCT | 33.9128 | 3' | CTGTGCGTGTGACAGCGGCT | 28.13 | 0.220334072 | 1.21 |  |
| hsa-miR-32 | TATTGCACATTACTAAGTTGCA | 5' | TATTGCACATTACTAAGTTG | 3.8537 | 5' | TATTGCACATTACTAAGTTG | 3.05 | 0.60736584 | 1.26 |  |
| hsa-miR-193b | AACTGGCCCTCAAAGTCCCGCT | 3' | AACTGGCCCTCAAAGTCC | 30.8299 | 3' | AACTGGCCCTCAAAGTCC | 24.40 | 0.148960573 | 1.26 |  |
| hsa-miR-122 | TGGAGTGTGACAATGGTGTTTG | 5' | TGGAGTGTGACAATGGTGTTTG | 3.083 | 5' | TGGAGTGTGACAATGGTGTTT | 2.37 | 0.610116524 | 1.30 |  |
| hsa-miR-1268 | CGGGCGTGGTGGTGGGGG | 5' | CGGGCGTGGTGGTGGGGG | 12.3319 | 5' | CGGGCGTGGTGGTGGGGG | 9.49 | 0.309698961 | 1.30 |  |
| hsa-miR-27b* | AGAGCTTAGCTGATTGGTGAAC | 5' | AGAGCTTAGCTGATTGGTGA | 37.7666 | 5' | AGAGCTTAGCTGATTGGTGA | 28.80 | 0.067001805 | 1.31 |  |
| hsa-miR-98 | TGAGGTAGTAAGTTGTATTGTT | 5' | TGAGGTAGTAAGTTGTATT | 117.1535 | 5' | TGAGGTAGTAAGTTGTATT | 89.12 | 0.0011387 | 1.31 |  |
| hsa-miR-101 | TACAGTACTGTGATAACTGAA | 3' | GTACAGTACTGTGATAACT | 1957.3107 | 3' | GTACAGTACTGTGATAACT | 1484.86 | 4.45E-41 | 1.32 |  |
| hsa-miR-423-3p | AGCTCGGTCTGAGGCCCCTCAGT | 3' | AGCTCGGTCTGAGGCCCCTCAGT | 127.1732 | 3' | AGCTCGGTCTGAGGCCCCTCAGT | 96.23 | 0.000559375 | 1.32 |  |
| hsa-miR-1284 | TCTATACAGACCCTGGCTTTTC | 5' | TCTATACAGACCCTGGCTTTTC | 2.6976 | 5' | TCTATACAGACCCTGGCTTTTC | 2.03 | 0.609681725 | 1.33 |  |
| hsa-miR-186 | CAAAGAATTCTCCTTTTGGGCT | 5' | CAAAGAATTCTCCTTTTGGGCTT | 32.7567 | 5' | CAAAGAATTCTCCTTTTGGGCTT | 24.40 | 0.065234748 | 1.34 |  |
| hsa-miR-23b | ATCACATTGCCAGGGATTACC | 3' | ATCACATTGCCAGGGATTACCAC | 122.1633 | 3' | ATCACATTGCCAGGGATTACCA | 90.47 | 0.000290316 | 1.35 |  |
| hsa-miR-132 | TAACAGTCTACAGCCATGGTCG | 3' | ACAGTCTACAGCCATGGTCG | 2.3122 | 3' | TAACAGTCTACAGCCATGGT | 1.69 | 0.607254833 | 1.36 |  |
| hsa-miR-21* | CAACACCAGTCGATGGGCTGT | 3' | CAACACCAGTCGATGGGCTGTCT | 2.3122 | 3' | CAACACCAGTCGATGGGCT | 1.69 | 0.607254833 | 1.36 |  |
| hsa-miR-29c | TAGCACCATTTGAAATCGGTTA | 3' | TAGCACCATTTGAAATCGGTTA | 395.7783 | 3' | TAGCACCATTTGAAATCGGTTA | 286.67 | 3.23E-12 | 1.38 |  |
| hsa-miR-590-5p | GAGCTTATTCATAAAAGTGCAG | 5' | GAGCTTATTCATAAAAGTGCAG | 4.2391 | 5' | GAGCTTATTCATAAAAGTGCAG | 3.05 | 0.463545591 | 1.39 |  |
| hsa-miR-664* | ACTGGCTAGGGAAAATGATTGGAT | 5' | ACTGGCTAGGGAAAATGATTGG | 8.4782 | 5' | ACTGGCTAGGGAAAATGATTGGA | 6.10 | 0.299275374 | 1.39 |  |
| hsa-miR-548k | AAAAGTACTTGCGGATTTTGCT | 5' | AAAAGTACTTGCGGATTTT | 3.8537 | 5' | AAAAGTACTTGCGGATTTTGCT | 2.71 | 0.458711788 | 1.42 |  |
| hsa-miR-125a-3p | ACAGGTGAGGTTCTTGGGAGCC | 3' | ACAGGTGAGGTTCTTGGGAGC | 1.9269 | 3' | ACAGGTGAGGTTCTTGGGA | 1.36 | 0.601738435 | 1.42 |  |
| hsa-miR-34c-5p | AGGCAGTGTAGTTAGCTGATTGC | 5' | TAGGCAGTGTAGTTAGCT | 1.9269 | 5' | AGGCAGTGTAGTTAGCTGATTG | 1.36 | 0.601738435 | 1.42 |  |
| hsa-miR-191 | CAACGGAATCCCAAAAGCAGCTG | 5' | CAACGGAATCCCAAAAGCAG | 19886.8005 | 5' | CAACGGAATCCCAAAAGCAGC | 13975.41 | 0 | 1.42 |  |
| hsa-miR-365 | TAATGCCCCTAAAAATCCTTAT | 3' | TAATGCCCCTAAAAATCCTTAT | 28.5176 | 3' | TAATGCCCCTAAAAATCCTTAT | 19.99 | 0.041181161 | 1.43 |  |
| hsa-miR-30c-1* | CTGGGAGAGGGTTGTTTACTCC | 3' | CTGGGAGAGGGTTGTTTACT | 35.8397 | 3' | CTGGGAGAGGGTTGTTTACT | 25.08 | 0.021393658 | 1.43 |  |
| hsa-miR-1278 | TAGTACTGTGCATATCATCTAT | 3' | TAGTACTGTGCATATCATCTAT | 26.5908 | 3' | TAGTACTGTGCATATCATCTAT | 18.30 | 0.038942468 | 1.45 |  |
| hsa-let-7a | TGAGGTAGTAGGTTGTATAGTT | 5' | TGAGGTAGTAGGTTGTATAGTT | 307802.9987 | 5' | TGAGGTAGTAGGTTGTATAGTT | 210817.75 | 0 | 1.46 |  |
| hsa-miR-224 | CAAGTCACTAGTGGTTCCGTT | 5' | CAAGTCACTAGTGGTTCCGTTTA | 18.4979 | 5' | CAAGTCACTAGTGGTTCCGTTT | 12.54 | 0.074388561 | 1.48 |  |
| hsa-miR-15b* | CGAATCATTATTTGCTGCTCTA | 3' | CGAATCATTATTTGCTGCT | 6.5513 | 3' | CGAATCATTATTTGCTGCTC | 4.41 | 0.281015371 | 1.49 |  |
| hsa-miR-130b | CAGTGCAATGATGAAAGGGCAT | 3' | CAGTGCAATGATGAAAGG | 120.2364 | 3' | CAGTGCAATGATGAAAGG | 79.63 | 1.60E-06 | 1.51 |  |
| hsa-miR-941 | CACCCGGCTGTGTGCACATGTGC | 5' | CACCCGGCTGTGTGCACATGTG | 1.5415 | 5' | CACCCGGCTGTGTGCACATGTGC | 1.02 | 0.591119658 | 1.52 |  |
| hsa-miR-454* | ACCCTATCAATATTGTCTCTGC | 5' | ACCCTATCAATATTGTCTC | 4.6245 | 5' | ACCCTATCAATATTGTCTC | 3.05 | 0.345785897 | 1.52 |  |
| hsa-let-7e* | CTATACGGCCTCCTAGCTTTCC | 3' | CTATACGGCCTCCTAGCTTT | 1.5415 | 3' | CTATACGGCCTCCTAGCTTTCC | 1.00 | 0.354631778 | 1.54 |  |
| hsa-miR-126* | CATTATTACTTTTGGTACGCG | 5' | CATTATTACTTTTGGTACGCG | 1.5415 | 3' | CTATACGGCCTCCTAGCTTTCC | 1.00 | 0.044845918 | 1.54 |  |
| hsa-miR-130a | CAGTGCAATGTTAAAAGGGCAT | 3' | CAGTGCAATGTTAAAAGG | 1.5415 | 3' | CAGTGCAATGTTAAAAGGGC | 1.00 | 0.354631778 | 1.54 |  |
| hsa-miR-141* | CATCTTCCAGTACAGTGTTGGA | 5' | CATCTTCCAGTACAGTGTT | 1.5415 | 3' | CAGTGCAATGTTAAAAGGGC | 1.00 | 0.044845918 | 1.54 |  |
| hsa-miR-19b-1* | AGTTTTGCAGGTTTGCATCCAGC | 5' | AGTTTTGCAGGTTTGCATCCAG | 1.5415 | 3' | CAGTGCAATGTTAAAAGGGC | 1.00 | 0.044845918 | 1.54 |  |
| hsa-miR-33b* | CAGTGCCTCGGCAGTGCAGCCC | 3' | CAGTGCCTCGGCAGTGCAGCC | 1.5415 | 3' | CAGTGCAATGTTAAAAGGGC | 1.00 | 0.044845918 | 1.54 |  |
| hsa-miR-378* | CTCCTGACTCCAGGTCCTGTGT | 5' | CTCCTGACTCCAGGTCCT | 1.5415 | 3' | CAGTGCAATGTTAAAAGGGC | 1.00 | 0.044845918 | 1.54 |  |
| hsa-miR-410 | AATATAACACAGATGGCCTGT | 3' | AATATAACACAGATGGCCTGT | 1.5415 | 3' | CAGTGCAATGTTAAAAGGGC | 1.00 | 0.044845918 | 1.54 |  |
| hsa-miR-488* | CCCAGATAATGGCACTCTCAA | 5' | CCAGATAATGGCACTCTCAAACA | 1.5415 | 3' | CAGTGCAATGTTAAAAGGGC | 1.00 | 0.044845918 | 1.54 |  |
| hsa-miR-942 | TCTTCTCTGTTTTGGCCATGTG | 5' | TCTTCTCTGTTTTGGCCATGTGT | 1.5415 | 3' | CAGTGCAATGTTAAAAGGGC | 1.00 | 0.044845918 | 1.54 |  |
| hsa-miR-193b* | CGGGGTTTTGAGGGCGAGATGA | 5' | CGGGGTTTTGAGGGCGAGATGA | 164.5544 | 5' | CGGGGTTTTGAGGGCGAGATGA | 105.38 | 1.72E-09 | 1.56 |  |
| hsa-miR-153 | TTGCATAGTCACAAAAGTGATC | 3' | TTGCATAGTCACAAAAGTGATCAT | 6.9367 | 3' | TTGCATAGTCACAAAAGTG | 4.41 | 0.211511564 | 1.57 |  |
| hsa-miR-15a | TAGCAGCACATAATGGTTTGTG | 5' | TAGCAGCACATAATGGTTT | 615.0557 | 5' | TAGCAGCACATAATGGTTT | 389.34 | 1.00E-32 | 1.58 |  |
| hsa-miR-23a | ATCACATTGCCAGGGATTTCC | 3' | ATCACATTGCCAGGGATTTCCA | 97.8848 | 3' | ATCACATTGCCAGGGATTTCCA | 60.99 | 9.79E-07 | 1.60 |  |
| hsa-miR-378 | ACTGGACTTGGAGTCAGAAGG | 3' | ACTGGACTTGGAGTCAGAAGGC | 3249.0818 | 3' | ACTGGACTTGGAGTCAGAAGGC | 1993.49 | 2.53E-185 | 1.63 |  |
| hsa-miR-26a | TTCAAGTAATCCAGGATAGGCT | 5' | TTCAAGTAATCCAGGATAGGCT | 302.518 | 5' | TTCAAGTAATCCAGGATAGGCT | 184.34 | 3.04E-19 | 1.64 |  |
| hsa-miR-203 | GTGAAATGTTTAGGACCACTAG | 3' | GTGAAATGTTTAGGACCACTAG | 101.3532 | 3' | GTGAAATGTTTAGGACCACTAG | 60.99 | 1.14E-07 | 1.66 |  |
| hsa-miR-140-3p | TACCACAGGGTAGAACCACGG | 3' | ACCACAGGGTAGAACCACGGA | 2010.1068 | 3' | ACCACAGGGTAGAACCACGGA | 1196.84 | 6.30E-128 | 1.68 |  |
| hsa-miR-92a-1* | AGGTTGGGATCGGTTGCAATGCT | 5' | AGGTTGGGATCGGTTGCAAT | 632.3975 | 5' | AGGTTGGGATCGGTTGCAATGCT | 364.95 | 9.18E-46 | 1.73 |  |
| hsa-miR-425* | ATCGGGAATGTCGTGTCCGCCC | 3' | CATCGGGAATGTCGTGTC | 8.8636 | 3' | ATCGGGAATGTCGTGTCC | 5.08 | 0.091821241 | 1.74 |  |
| hsa-miR-1304 | TTTGAGGCTACAGTGAGATGTG | 5' | CGGTTTGAGGCTACAGTGAG | 4.2391 | 5' | TTTGAGGCTACAGTGAGATGTG | 2.37 | 0.230141065 | 1.79 |  |
| hsa-miR-548l | AAAAGTATTTGCGGGTTTTGTC | 5' | AAAAGTATTTGCGGGTTTTG | 3.083 | 5' | AAAAGTATTTGCGGGTTTTG | 1.69 | 0.296547995 | 1.82 |  |
| hsa-miR-500* | ATGCACCTGGGCAAGGATTCTG | 3' | ATGCACCTGGGCAAGGATT | 191.5305 | 3' | ATGCACCTGGGCAAGGATT | 104.37 | 1.78E-17 | 1.84 |  |
| hsa-miR-502-3p | AATGCACCTGGGCAAGGATTCA | 3' | ATGCACCTGGGCAAGGATT | 191.5305 | 3' | ATGCACCTGGGCAAGGATT | 104.03 | 1.27E-17 | 1.84 |  |
| hsa-miR-374a* | CTTATCAGATTGTATTGTAATT | 3' | CTTATCAGATTGTATTGTAATT | 18.4979 | 3' | CTTATCAGATTGTATTGTAATT | 9.83 | 0.006383407 | 1.88 |  |
| hsa-miR-1274b | TCCCTGTTCGGGCGCCA | 5' | GTCCCTGTTCGGGCGCCA | 1.9269 | 5' | GTCCCTGTTCGGGCGCCA | 1.00 | 0.087974057 | 1.93 |  |
| hsa-miR-25* | AGGCGGAGACTTGGGCAATTG | 5' | AGGCGGAGACTTGGGCAATTGCT | 555.7082 | 5' | AGGCGGAGACTTGGGCAATTGCT | 287.69 | 2.45E-54 | 1.93 |  |
| hsa-miR-1285 | TCTGGGCAACAAAGTGAGACCT | 3' | TCTGGGCAACAAAGTGAGAC | 13.8734 | 3' | TCTGGGCAACAAAGTGAGAC | 7.12 | 0.013601998 | 1.95 |  |
| hsa-miR-215 | ATGACCTATGAATTGACAGAC | 5' | ATGACCTATGAATTGACAGACA | 5.3952 | 5' | ATGACCTATGAATTGACAGACA | 2.71 | 0.117849641 | 1.99 |  |
| hsa-miR-576-5p | ATTCTAATTTCTCCACGTCTTT | 5' | ATTCTAATTTCTCCACGTCTTT | 10.4051 | 5' | ATTCTAATTTCTCCACGTCTTT | 5.08 | 0.023795805 | 2.05 |  |
| hsa-miR-505 | CGTCAACACTTGCTGGTTTCCT | 3' | TCAACACTTGCTGGTTTCCT | 4.2391 | 3' | CAACACTTGCTGGTTTCC | 2.03 | 0.145517834 | 2.09 |  |
| hsa-miR-505* | GGGAGCCAGGAAGTATTGATGT | 5' | GGGAGCCAGGAAGTATTGATGTT | 10.0197 | 5' | GGGAGCCAGGAAGTATTGATGTT | 4.74 | 0.021690244 | 2.11 |  |
| hsa-miR-29b | TAGCACCATTTGAAATCAGTGTT | 3' | TAGCACCATTTGAAATCAGT | 51.2546 | 3' | TAGCACCATTTGAAATCAGT | 24.06 | 1.30E-07 | 2.13 | ** |
| hsa-miR-15b | TAGCAGCACATCATGGTTTACA | 5' | TAGCAGCACATCATGGTTTACA | 2047.1026 | 5' | TAGCAGCACATCATGGTTTACA | 944.05 | 3.77E-254 | 2.17 | ** |
| hsa-miR-106b | TAAAGTGCTGACAGTGCAGAT | 5' | TAAAGTGCTGACAGTGCAGATA | 611.5873 | 5' | TAAAGTGCTGACAGTGCAGATA | 281.59 | 1.47E-77 | 2.17 | ** |
| hsa-miR-452 | AACTGTTTGCAGAGGAAACTGA | 5' | AACTGTTTGCAGAGGAAACT | 186.5206 | 5' | AACTGTTTGCAGAGGAAACT | 85.05 | 2.62E-25 | 2.19 | ** |
| hsa-miR-21 | TAGCTTATCAGACTGATGTTGA | 5' | TAGCTTATCAGACTGATGTT | 1687.9348 | 5' | TAGCTTATCAGACTGATGTT | 757.68 | 9.23E-222 | 2.23 | ** |
| hsa-miR-16 | TAGCAGCACGTAAATATTGGCG | 5' | TAGCAGCACGTAAATATTG | 3034.8142 | 5' | TAGCAGCACGTAAATATTG | 1362.20 | 0 | 2.23 | ** |
| hsa-miR-1260 | ATCCCACCTCTGCCACCA | 5' | ATCCCACCTCTGCCACCA | 4.6245 | 5' | ATCCCACCTCTGCCACCA | 2.03 | 0.096154283 | 2.27 |  |
| hsa-miR-454 | TAGTGCAATATTGCTTATAGGGT | 3' | TAGTGCAATATTGCTTATA | 4.6245 | 3' | TAGTGCAATATTGCTTATA | 2.03 | 0.096154283 | 2.27 |  |
| hsa-miR-17 | CAAAGTGCTTACAGTGCAGGTAG | 5' | CAAAGTGCTTACAGTGCAGGTAG | 225.8287 | 5' | CAAAGTGCTTACAGTGCAGGTAG | 97.93 | 2.71E-33 | 2.31 | ** |
| hsa-miR-150 | TCTCCCAACCCTTGTACCAGTG | 5' | TCTCCCAACCCTTGTACCA | 2.3122 | 5' | TCTCCCAACCCTTGTACCAG | 1.00 | 0.124219855 | 2.31 |  |
| hsa-miR-590-3p | TAATTTTATGTATAAGCTAGT | 3' | TAATTTTATGTATAAGCT | 2.3122 | 3' | GTAATTTTATGTATAAGCTAG | 1.00 | 0.124219855 | 2.31 |  |
| hsa-miR-497 | CAGCAGCACACTGTGGTTTGT | 5' | CAGCAGCACACTGTGGTTTGT | 56.6499 | 5' | CAGCAGCACACTGTGGTTTGTA | 23.38 | 3.13E-10 | 2.42 | ** |
| hsa-miR-532-5p | CATGCCTTGAGTGTAGGACCGT | 5' | CATGCCTTGAGTGTAGGACCGT | 156.4615 | 5' | CATGCCTTGAGTGTAGGACCGT | 64.04 | 5.30E-26 | 2.44 | ** |
| hsa-miR-20a | TAAAGTGCTTATAGTGCAGGTAG | 5' | TAAAGTGCTTATAGTGCAGGTAG | 70.9087 | 5' | TAAAGTGCTTATAGTGCAGGTA | 28.46 | 5.43E-13 | 2.49 | ** |
| hsa-miR-582-3p | TAACTGGTTGAACAACTGAACC | 3' | TAACTGGTTGAACAACTGAA | 3.4684 | 3' | TAACTGGTTGAACAACTGAA | 1.36 | 0.112990888 | 2.56 |  |
| hsa-miR-200a | TAACACTGTCTGGTAACGATGT | 3' | TAACACTGTCTGGTAACGAT | 518.7124 | 3' | TAACACTGTCTGGTAACGAT | 200.26 | 1.51E-90 | 2.59 | ** |
| hsa-miR-484 | TCAGGCTCAGTCCCCTCCCGAT | 5' | TCAGGCTCAGTCCCCTCCCGAT | 2.6976 | 5' | TCAGGCTCAGTCCCCTCCCGATAA | 1.02 | 0.154019401 | 2.65 |  |
| hsa-miR-28-5p | AAGGAGCTCACAGTCTATTGAG | 5' | AAGGAGCTCACAGTCTATTGAG | 17.3418 | 5' | AAGGAGCTCACAGTCTATTGAG | 6.44 | 0.000156516 | 2.69 | ** |
| hsa-let-7c* | TAGAGTTACACCCTGGGAGTTA | 5' | TAGAGTTACACCCTGGGAGTTAA | 2.6976 | 5' | TAGAGTTACACCCTGGGAGTTA | 1.00 | 0.070969744 | 2.70 |  |
| hsa-miR-889 | TTAATATCGGACAACCATTGT | 3' | TTAATATCGGACAACCATTGT | 2.6976 | 3' | TTAATATCGGACAACCATT | 1.00 | 0.070969744 | 2.70 |  |
| hsa-miR-193a-3p | AACTGGCCTACAAAGTCCCAGT | 3' | AACTGGCCTACAAAGTCC | 302.518 | 3' | AACTGGCCTACAAAGTCCCA | 111.82 | 3.24E-57 | 2.71 | ** |
| hsa-miR-940 | AAGGCAGGGCCCCCGCTCCCC | 3' | AAGGCAGGGCCCCCGCTCCC | 11.1758 | 3' | AAGGCAGGGCCCCCGCTCCC | 4.07 | 0.00214183 | 2.75 | ** |
| hsa-miR-532-3p | CCTCCCACACCCAAGGCTTGCA | 3' | CCTCCCACACCCAAGGCTTGCA | 33.9128 | 3' | CCTCCCACACCCAAGGCTTGCA | 12.20 | 5.62E-08 | 2.78 | ** |
| hsa-miR-425 | AATGACACGATCACTCCCGTTGA | 5' | AATGACACGATCACTCCCGTTGA | 22.737 | 5' | AATGACACGATCACTCCCGTTGAG | 8.13 | 8.30E-06 | 2.80 | ** |
| hsa-miR-195 | TAGCAGCACAGAAATATTGGC | 5' | TAGCAGCACAGAAATATTG | 359.9386 | 5' | TAGCAGCACAGAAATATTG | 127.07 | 2.31E-72 | 2.83 | ** |
| hsa-miR-29a | TAGCACCATCTGAAATCGGTTA | 3' | TAGCACCATCTGAAATCGGTTA | 4775.9305 | 3' | TAGCACCATCTGAAATCGGTTA | 1682.75 | 0 | 2.84 | ** |
| hsa-miR-1975 | CCCCCACAACCGCGCTTGACTAGCT | 3' | CCCCCCACAACCGCGCTTGACTAGC | 15.4149 | 3' | CCCCCACAACCGCGCTTGACTAGCT | 5.42 | 0.000211128 | 2.84 | ** |
| hsa-miR-22 | AAGCTGCCAGTTGAAGAACTGT | 3' | AAGCTGCCAGTTGAAGAA | 97.4994 | 3' | AAGCTGCCAGTTGAAGAA | 34.22 | 5.82E-21 | 2.85 | ** |
| hsa-miR-200c | TAATACTGCCGGGTAATGATGGA | 3' | TAATACTGCCGGGTAATGATGGA | 1619.7237 | 3' | TAATACTGCCGGGTAATGATGG | 567.58 | 3.16202013338398e-322 | 2.85 | ** |
| hsa-miR-19b | TGTGCAAATCCATGCAAAACTGA | 3' | TGTGCAAATCCATGCAAAACT | 14.6442 | 3' | TGTGCAAATCCATGCAAAACT | 5.08 | 0.000268735 | 2.88 | ** |
| hsa-miR-22* | AGTTCTTCAGTGGCAAGCTTTA | 5' | AGTTCTTCAGTGGCAAGCTTT | 11.1758 | 5' | AGTTCTTCAGTGGCAAGCTT | 3.73 | 0.001107135 | 3.00 | ** |
| hsa-miR-219-1-3p | AGAGTTGAGTCTGGACGTCCCG | 3' | AGAGTTGAGTCTGGACGTCCCG | 3.083 | 3' | AGAGTTGAGTCTGGACGTCCCG | 1.02 | 0.093255379 | 3.03 |  |
| hsa-miR-424 | CAGCAGCAATTCATGTTTTGAA | 5' | CAGCAGCAATTCATGTTTTGA | 141.0466 | 5' | CAGCAGCAATTCATGTTTTGA | 46.08 | 1.76E-32 | 3.06 | ** |
| hsa-miR-93 | CAAAGTGCTGTTCGTGCAGGTAG | 5' | CAAAGTGCTGTTCGTGCAGGTA | 1282.1368 | 5' | CAAAGTGCTGTTCGTGCAGGTA | 411.71 | 4.32E-287 | 3.11 | ** |
| hsa-miR-577 | TAGATAAAATATTGGTACCTG | 5' | GTAGATAAAATATTGGTAC | 8.4782 | 5' | GTAGATAAAATATTGGTACCTG | 2.71 | 0.003630552 | 3.13 |  |
| hsa-miR-29b-2* | CTGGTTTCACATGGTGGCTTAG | 5' | CTGGTTTCACATGGTGGCTTA | 4.2391 | 5' | GCTGGTTTCACATGGTGGCTTA | 1.36 | 0.042651253 | 3.13 |  |
| hsa-miR-429 | TAATACTGTCTGGTAAAACCGT | 3' | TAATACTGTCTGGTAAAACCGT | 45.8594 | 3' | TAATACTGTCTGGTAAAACCGT | 13.55 | 6.94E-13 | 3.38 | ** |
| hsa-miR-96 | TTTGGCACTAGCACATTTTTGCT | 5' | TTTGGCACTAGCACATTTTTGC | 33.5275 | 5' | TTTGGCACTAGCACATTTTTGC | 9.83 | 7.16E-10 | 3.41 | ** |
| hsa-miR-375 | TTTGTTCGTTCGGCTCGCGTGA | 3' | TTTGTTCGTTCGGCTCGCGTG | 43.1618 | 3' | TTTGTTCGTTCGGCTCGCGTG | 12.54 | 2.00E-12 | 3.44 | ** |
| hsa-miR-139-5p | TCTACAGTGCACGTGTCTCCAG | 5' | TCTACAGTGCACGTGTCTCCAGT | 8.8636 | 5' | TCTACAGTGCACGTGTCTCCA | 2.37 | 0.001004689 | 3.74 |  |
| hsa-miR-324-5p | CGCATCCCCTAGGGCATTGGTGT | 5' | CGCATCCCCTAGGGCATT | 62.0451 | 5' | CGCATCCCCTAGGGCATT | 16.27 | 4.80E-19 | 3.81 | ** |
| hsa-miR-190b | TGATATGTTTGATATTGGGTT | 5' | TGATATGTTTGATATTGGGTTG | 3.8537 | 5' | TGATATGTTTGATATTGGGTTGT | 1.00 | 0.012016884 | 3.85 |  |
| hsa-miR-556-3p | ATATTACCATTAGCTCATCTTT | 3' | ATATTACCATTAGCTCATCTT | 5.3952 | 3' | ATATTACCATTAGCTCATCTT | 1.36 | 0.008810418 | 3.98 |  |
| hsa-miR-141 | TAACACTGTCTGGTAAAGATGG | 3' | TAACACTGTCTGGTAAAG | 78.6161 | 3' | TAACACTGTCTGGTAAAGATG | 19.31 | 3.61E-25 | 4.07 | ** |
| hsa-miR-589* | TCAGAACAAATGCCGGTTCCCAGA | 3' | TCAGAACAAATGCCGGTTCCCAGA | 11.9466 | 3' | TCAGAACAAATGCCGGTTCCCAGA | 2.71 | 3.46E-05 | 4.41 | ** |
| hsa-miR-200b* | CATCTTACTGGGCAGCATTGGA | 5' | CATCTTACTGGGCAGCATT | 344.5237 | 5' | CATCTTACTGGGCAGCATT | 76.24 | 1.02E-114 | 4.52 | ** |
| hsa-miR-361-5p | TTATCAGAATCTCCAGGGGTAC | 5' | TTATCAGAATCTCCAGGGGTAC | 6.166 | 5' | TTATCAGAATCTCCAGGGGTAC | 1.36 | 0.002901443 | 4.55 |  |
| hsa-miR-9* | ATAAAGCTAGATAACCGAAAGT | 3' | TAAAGCTAGATAACCGAAAGT | 44.7033 | 3' | TAAAGCTAGATAACCGAAAGT | 9.15 | 3.27E-17 | 4.89 | ** |
| hsa-miR-186* | GCCCAAAGGTGAATTTTTTGGG | 3' | GCCCAAAGGTGAATTTTTT | 5.0099 | 3' | TAAAGCTAGATAACCGAAAGT | 1.00 | 4.82E-05 | 5.01 |  |
| hsa-miR-652 | AATGGCGCCACTAGGGTTGTG | 3' | AATGGCGCCACTAGGGTT | 5.0099 | 3' | AATGGCGCCACTAGGGTT | 1.00 | 0.001839953 | 5.01 |  |
| hsa-miR-339-5p | TCCCTGTCCTCCAGGAGCTCACG | 5' | TCCCTGTCCTCCAGGAGCTCACG | 10.4051 | 5' | TCCCTGTCCTCCAGGAGC | 2.03 | 4.30E-05 | 5.12 |  |
| hsa-miR-331-3p | GCCCCTGGGCCTATCCTAGAA | 3' | GCCCCTGGGCCTATCCTAGAA | 41.2349 | 3' | GCCCCTGGGCCTATCCTAGAA | 7.79 | 7.17E-17 | 5.29 | ** |
| hsa-miR-1973 | ACCGTGCAAAGGTAGCATA | 3' | ACCGTGCAAAGGTAGCATA | 7.3221 | 3' | ACCGTGCAAAGGTAGCATA | 1.36 | 0.000512194 | 5.40 |  |
| hsa-miR-200b | TAATACTGCCTGGTAATGATGA | 3' | TAATACTGCCTGGTAATGATGAC | 284.0201 | 3' | TAATACTGCCTGGTAATGATGAC | 51.84 | 1.09E-109 | 5.48 | ** |
| hsa-miR-362-3p | AACACACCTATTCAAGGATTCA | 3' | AACACACCTATTCAAGGATTCA | 5.7806 | 3' | AACACACCTATTCAAGGATTCA | 1.00 | 0.00010037 | 5.78 |  |
| hsa-miR-200c* | CGTCTTACCCAGCAGTGTTTGG | 5' | CGTCTTACCCAGCAGTGTTTGG | 6.166 | 3' | AACACACCTATTCAAGGATTCA | 1.00 | 4.94E-06 | 6.17 |  |
| hsa-miR-96* | AATCATGTGCAGTGCCAATATG | 3' | CAATCATGTGCAGTGCCAATAT | 16.1857 | 3' | CAATCATGTGCAGTGCCAATA | 2.37 | 1.83E-08 | 6.82 | ** |
| hsa-miR-106a | AAAAGTGCTTACAGTGCAGGTAG | 5' | AAAGTGCTTACAGTGCAGGTAG | 11.9466 | 5' | AAAGTGCTTACAGTGCAGG | 1.69 | 1.19E-06 | 7.05 | ** |
| hsa-miR-29c* | TGACCGATTTCTCCTGGTGTTC | 5' | ACCGATTTCTCCTGGTGTTCAG | 7.3221 | 5' | GACCGATTTCTCCTGGTGTT | 1.00 | 5.89E-06 | 7.32 |  |
| hsa-miR-362-5p | AATCCTTGGAACCTAGGTGTGAGT | 5' | AATCCTTGGAACCTAGGTGTGAGT | 11.1758 | 5' | AATCCTTGGAACCTAGGTGTGAGT | 1.36 | 1.06E-06 | 8.25 | ** |
| hsa-miR-301b | CAGTGCAATGATATTGTCAAAGC | 3' | CAGTGCAATGATATTGTCAAAGCA | 13.1027 | 3' | CAGTGCAATGATATTGTCAAAGC | 1.36 | 4.15E-08 | 9.67 | ** |
| hsa-miR-1277 | TACGTAGATATATATGTATTTT | 3' | TACGTAGATATATATGTATTTT | 45.474 | 3' | TACGTAGATATATATGTATTTT | 4.41 | 1.09E-25 | 10.32 | ** |
| hsa-miR-664 | TATTCATTTATCCCCAGCCTACA | 3' | TATTCATTTATCCCCAGCCTACA | 18.4979 | 3' | TATTCATTTATCCCCAGCCTACAA | 1.00 | 3.72E-15 | 18.50 | ** |
| hsa-miR-301a | CAGTGCAATAGTATTGTCAAAGC | 3' | CAGTGCAATAGTATTGTCAAAGCAT | 75.1478 | 3' | CAGTGCAATAGTATTGTCAAAGC | 3.05 | 2.34E-52 | 24.64 | ** |
