# Supplementary material for: Characterization of the Small RNA Transcriptomes of Androgen Dependent and Independent Prostate Cancer Cell Line by Deep Sequencing
Source: PLoS One. 2010 Nov 30;5(11):e15519. doi: 10.1371/journal.pone.0015519 (PMC2994876; doi:10.1371/journal.pone.0015519)
Supplement: Table S2 — The novel miRNAs identified in LNCaP and LNCaP-AI libraries. (DOC) [file pone.0015519.s002.doc]

**Table S2. The novel miRNAs identified in LNCaP and LNCaP-AI libraries**

| miRNA ID | Sequence | Length (nt) | Genomic location | 5'/3'  arm | Precursor  length (nt) | MFE | Relative count  in LNCaP-AI | Relative count  in LNCaP | qRT-PCR  validation |
| --- | --- | --- | --- | --- | --- | --- | --- | --- | --- |
| hsa-novel-miR-01 | TCGGGCGGGAGTGGTGGCTTTT | 22 | chr6:28918819:28918903:+ | 3' | 85 | -22.2 | 816.64 | 277.47 | * |
| hsa-novel-miR-02 | ACTGACAGGAGAGCATTTTGA | 21 | chr5:89312448:89312527:- | 3' | 80 | -39.3 | 45.41. | 0 | * |
| hsa-novel-miR-03 | TCTCAGGAGTAAAGACAGAGTT | 22 | chr11:70718384:70718464:- | 3' | 81 | -49.2 | 21.69 | 0 | * |
| hsa-novel-miR-04 | GCAAATGATGTGAGAGATTC | 20 | chr6:168343859:168343952:+ | 3' | 94 | -20.2 | 11.52 | 0 | * |
| hsa-novel-miR-05 | AAAAGCTGGGTTGAGAGGGTAA | 22 | chr18:21901636:21901711:+ | 3' | 76 | -32.6 | 11.18 | 4.62 | * |
| hsa-novel-miR-06 | ACTGGACTTGGAGTCAGAAGA | 21 | chr10:132760891:132760988:- | 3' | 98 | -34.9 | 10.17 | 10.02 | * |
| hsa-novel-miR-07 | TCTGATGATGATGATGGTGCT | 21 | chr15:89826590:89826677:+ | 5' | 88 | -18.2 | 0 | 24.66 | * |
| hsa-novel-miR-08 | CAAAATGATGAGGTACCTGATA | 22 | chr20:3194751:3194835:+ | 5' | 85 | -20.4 | 0 | 73.22 | * |
| hsa-novel-miR-09 | ACCCCAGGATGCCAGCATAGTT | 22 | chr5:138611933:138612022:+ | 5' | 90 | -32.2 | 0 | 19.65 | * |
| hsa-novel-miR-10 | TAGCTCTGATGATGGTGGTTTCT | 23 | chr5:176878879:176878970:- | 3' | 92 | -23.2 | 0 | 11.18 | * |
| hsa-novel-miR-11 | TTATTTATTATATTTTATATT | 21 | chr13:81859787:81859884:+ | 3' | 98 | -20.8 | 393.75 | 546.07 |  |
| hsa-novel-miR-12 | AGGGAACACAGTACGGCTTG | 20 | chr12:125424202:125424294:- | 5' | 93 | -30.4 | 9.49 | 0 |  |
| hsa-novel-miR-13 | TTGTGGAAACAATGGTACGGCA | 22 | chr15:45493361:45493452:+ | 3' | 92 | -28.7 | 8.13 | 5.40 |  |
| hsa-novel-miR-14 | GGAGGAACCTTGGAGCTTCGGCA | 23 | chr22:31556037:31556127:- | 3' | 91 | -45.3 | 5.08 | 2.31 |  |
| hsa-novel-miR-15 | GCTGCACCGGAGACTGGGTAA | 21 | chr2:207647959:207648031:+ | 3' | 73 | -67.4 | 5.08 | 3.85 |  |
| hsa-novel-miR-16 | CAGGCAGTGACTGTTCAGACGTC | 23 | chr1:98510819:98510895:- | 5' | 77 | -38.9 | 4.41 | 0 |  |
| hsa-novel-miR-17 | CTGTCCTAAGGTTGTTGAGTT | 21 | chrX:69242704:69242779:+ | 5' | 76 | -33.7 | 4.41 | 6.94 |  |
| hsa-novel-miR-18 | TCGGGCGGGCGGGAGGTGCA | 20 | chr11:76092079:76092159:- | 5' | 81 | -46.2 | 4.07 | 0 |  |
| hsa-novel-miR-19 | AAGGGAGGAGGAGCGGAGGG | 20 | chr17:77680983:77681059:- | 5' | 77 | -46.9 | 3.73 | 0 |  |
| hsa-novel-miR-20 | ATCAGGGCTTGTGGAATGGGAAG | 23 | chr2:97464015:97464091:+ | 5' | 77 | -47.3 | 3.39 | 0 |  |
| hsa-novel-miR-21 | AGACAGGTGGCCGGTTAGCT | 20 | chr6:26554332:26554408:+ | 5' | 77 | -22.3 | 3.39 | 0 |  |
| hsa-novel-miR-22 | TGGGAGGAACAAGTATGCATT | 21 | chr11:16984501:16984581:- | 3' | 81 | -27.1 | 3.05 | 4.62 |  |
| hsa-novel-miR-23 | AGGGAAGGAGGCTTGGTCTTAG | 22 | chr19:4932689:4932764:+ | 5' | 76 | -30.14 | 2.71 | 0 |  |
| hsa-novel-miR-24 | TCTGGTGAGTAGTGCATGGCT | 21 | chrX:83658918:83658998:+ | 3' | 81 | -19.7 | 2.71 | 0 |  |
| hsa-novel-miR-25 | TGTTGTACTTTTTTTTTTGTTC | 22 | chr13:50570556:50570632:- | 5' | 77 | -27 | 2.03 | 0 |  |
| hsa-novel-miR-26 | TCGGGGAGATGAGAGACGTG | 20 | chr6:42071607:42071696:- | 5' | 90 | -31.9 | 2.03 | 0 |  |
| hsa-novel-miR-27 | GCAAAGTGATGAGTAATACT | 20 | chr7:98479272:98479351:+ | 3' | 80 | -25.5 | 2.03 | 0 |  |
| hsa-novel-miR-28 | ACAAGGAAGGACAAGAGGTGT | 21 | chr10:687634:687712:- | 5' | 79 | -49.7 | 1.69 | 3.85 |  |
| hsa-novel-miR-29 | AAGGATGAGGCAGGAAAGATT | 21 | chr11:34646640:34646726:+ | 5' | 87 | -22.2 | 1.69 | 0 |  |
| hsa-novel-miR-30 | CAGCATACCATGGGAGCATCT | 21 | chr14:74963181:74963255:+ | 5' | 75 | -19.8 | 1.69 | 0 |  |
| hsa-novel-miR-31 | AGATGTATGGAATCTGTATAT | 21 | chr14:28102409:28102485:- | 5' | 77 | -34 | 1.69 | 0 |  |
| hsa-novel-miR-32 | AAGGAAAGAGAAACAAGCATTAAA | 24 | chr15:41641711:41641794:+ | 5' | 84 | -27.1 | 1.69 | 0 |  |
| hsa-novel-miR-33 | ACTGGCAAAAGGGTTTAGAA | 20 | chr16:50830166:50830246:+ | 5' | 81 | -20.34 | 1.69 | 0 |  |
| hsa-novel-miR-34 | AGGAGGAATGGTGGCATCTTT | 21 | chr19:48155081:48155165:+ | 5' | 85 | -34.8 | 1.69 | 0 |  |
| hsa-novel-miR-35 | ATAAATGTTGGGCATGGCAAT | 21 | chr6:160838656:160838720:+ | 5' | 65 | -19.2 | 1.69 | 0 |  |
| hsa-novel-miR-36 | ATATTTATTATATTTTATATT | 21 | chr12:9215293:9215382:+ | 5' | 90 | -21.5 | 0 | 2.70 |  |
| hsa-novel-miR-37 | AGAAGGGGTGAAATTTAAACGT | 22 | chr16:14995365:14995447:+ | 3' | 83 | -58.7 | 0 | 3.85 |  |
| hsa-novel-miR-38 | TCTGGGCACAGGCGGATGGACAG | 23 | chr16:88535341:88535424:+ | 5' | 84 | -46.7 | 0 | 1.93 |  |
| hsa-novel-miR-39 | CCGTGTTTCCCCCACGCTTT | 20 | chr17:8090489:8090581:+ | 3' | 93 | -33.3 | 0 | 3.47 |  |
| hsa-novel-miR-40 | TCTGGAGGTGGAAGGAAAGGATT | 23 | chr1:120267343:120267436:+ | 5' | 94 | -31 | 0 | 5.01 |  |
| hsa-novel-miR-41 | TCTGGTATGTAGTAGGTAATA | 21 | chr2:169439453:169439529:+ | 5' | 77 | -28.7 | 0 | 1.93 |  |
| hsa-novel-miR-42 | ATGGGGACAGGGATCAGCATGG | 22 | chr2:219206629:219206708:+ | 5' | 80 | -36.7 | 0 | 2.70 |  |
| hsa-novel-miR-43 | AGAATTGCGTTTGGACAATCAG | 22 | chr6:33175621:33175704:- | 3' | 84 | -40.8 | 0 | 1.93 |  |

* indicates that the miRNA has been selected to perform qRT-PCR validation.
