# Supplementary material for: Characterization of the Small RNA Transcriptomes of Androgen Dependent and Independent Prostate Cancer Cell Line by Deep Sequencing
Source: PLoS One. 2010 Nov 30;5(11):e15519. doi: 10.1371/journal.pone.0015519 (PMC2994876; doi:10.1371/journal.pone.0015519)
Supplement: Table S3 — The GO term predicted targets of differentially expressed miRNAs. (DOC) [file pone.0015519.s003.doc]

**Table S3. The GO term predicted targets of differentially expressed miRNAs**

| **GO ID** | **GO term** | **Gene count** | **Percentage (%)** | **P-value** | **Benjamini correction** |
| --- | --- | --- | --- | --- | --- |
| GO:0006464 | protein modification process | 752 | 13.1 | 2.6E-29 | 6.6E-26 |
| GO:0006357 | regulation of transcription from RNA polymerase II promoter | 248 | 4.3 | 4.3E-24 | 5.6E-21 |
| GO:0019219 | regulation of nucleobase, nucleoside, nucleotide and nucleic acid metabolic process | 1004 | 17.5 | 1.7E-21 | 1.5E-18 |
| GO:0045449 | regulation of transcription | 975 | 17.0 | 9.0E-20 | 5.9E-17 |
| GO:0006355 | regulation of transcription, DNA-dependent | 915 | 16.0 | 8.9E-19 | 4.6E-16 |
| GO:0006351 | transcription, DNA-dependent | 928 | 16.2 | 6.9E-18 | 3.0E-15 |
| GO:0032774 | RNA biosynthetic process | 928 | 16.2 | 1.0E-17 | 3.8E-15 |
| GO:0016481 | negative regulation of transcription | 158 | 2.8 | 5.5E-16 | 1.8E-13 |
| GO:0007167 | enzyme linked receptor protein signaling pathway | 150 | 2.6 | 3.3E-15 | 9.3E-13 |
| GO:0031324 | negative regulation of cellular metabolic process | 190 | 3.3 | 5.0E-15 | 1.3E-12 |
| GO:0045893 | positive regulation of transcription, DNA-dependent | 140 | 2.4 | 9.0E-15 | 2.1E-12 |
| GO:0045935 | positive regulation of nucleobase, nucleoside, nucleotide and nucleic acid metabolic process | 169 | 3.0 | 1.2E-14 | 2.6E-12 |
| GO:0045941 | positive regulation of transcription | 164 | 2.9 | 3.0E-14 | 6.0E-12 |
| GO:0022008 | neurogenesis | 158 | 2.8 | 4.5E-14 | 8.3E-12 |
| GO:0045934 | negative regulation of nucleobase, nucleoside, nucleotide and nucleic acid metabolic process | 164 | 2.9 | 5.9E-14 | 1.0E-11 |
| GO:0000902 | cell morphogenesis | 229 | 4.0 | 8.0E-14 | 1.3E-11 |
| GO:0007417 | central nervous system development | 136 | 2.4 | 1.3E-13 | 2.0E-11 |
| GO:0031325 | positive regulation of cellular metabolic process | 195 | 3.4 | 6.0E-13 | 8.6E-11 |
| GO:0007264 | small GTPase mediated signal transduction | 225 | 3.9 | 7.5E-13 | 1.0E-10 |
