# Supplementary material for: Characterization of the Small RNA Transcriptomes of Androgen Dependent and Independent Prostate Cancer Cell Line by Deep Sequencing
Source: PLoS One. 2010 Nov 30;5(11):e15519. doi: 10.1371/journal.pone.0015519 (PMC2994876; doi:10.1371/journal.pone.0015519)
Supplement: Table S4 — The KEGG term of predicted targets of differentially expressed miRNAs. (DOC) [file pone.0015519.s004.doc]

**Table S4. The KEGG term of predicted targets of differentially expressed miRNAs**

| **KEGG term** | **Gene count** | **Percentage (%)** | **P-value** | **Benjamini correction** |
| --- | --- | --- | --- | --- |
| Axon guidance | 79 | 1.4 | 3.5E-10 | 7.1E-08 |
| Focal adhesion | 107 | 1.9 | 1.2E-09 | 1.2E-07 |
| MAPK signaling pathway | 130 | 2.3 | 5.2E-09 | 3.5E-07 |
| Wnt signaling pathway | 84 | 1.5 | 1.2E-08 | 6.0E-07 |
| Renal cell carcinoma | 45 | 0.8 | 3.4E-08 | 1.4E-06 |
| TGF-beta signaling pathway | 54 | 0.9 | 3.0E-07 | 1.0E-05 |
| Long-term potentiation | 42 | 0.7 | 5.2E-07 | 1.5E-05 |
| Regulation of actin cytoskeleton | 105 | 1.8 | 8.2E-07 | 2.1E-05 |
| Melanogenesis | 56 | 1 | 1.5E-06 | 3.5E-05 |
| Adherens junction | 44 | 0.8 | 6.5E-06 | 1.3E-04 |
| Colorectal cancer | 47 | 0.8 | 4.0E-05 | 7.2E-04 |
| GnRH signaling pathway | 50 | 0.9 | 8.1E-05 | 1.4E-03 |
| Pancreatic cancer | 41 | 0.7 | 8.7E-05 | 1.3E-03 |
| ErbB signaling pathway | 46 | 0.8 | 9.7E-05 | 1.4E-03 |
